# Macropinocytosis requires Gal-3 in a subset of patient-derived glioblastoma stem cells

**DOI:** 10.1038/s42003-021-02258-z

**Published:** 2021-06-10

**Authors:** Laetitia Seguin, Soline Odouard, Francesca Corlazzoli, Sarah Al Haddad, Laurine Moindrot, Marta Calvo Tardón, Mayra Yebra, Alexey Koval, Eliana Marinari, Viviane Bes, Alexandre Guérin, Mathilde Allard, Sten Ilmjärv, Vladimir L. Katanaev, Paul R. Walker, Karl-Heinz Krause, Valérie Dutoit, Jann N. Sarkaria, Pierre-Yves Dietrich, Érika Cosset

**Affiliations:** 1 University Côte d’Azur, CNRS UMR7284, INSERM U1081, Institute for Research on Cancer and Aging (IRCAN), Nice, France; 2 Laboratory of Tumor Immunology, Department of Oncology, Center for Translational Research in Onco-Hematology, Swiss Cancer Center Léman (SCCL), Geneva University Hospitals, University of Geneva, Geneva, Switzerland; 3 Laboratory of Immunobiology of brain tumors, Center for Translational Research in Onco-Hematology, Geneva University Hospitals, and University of Geneva, Geneva, Switzerland; 4 Department of Surgery, Moores Cancer Center, University of California San Diego, La Jolla, CA USA; 5 Department of Cell Physiology and Metabolism, Medical School, University of Geneva, Geneva, Switzerland; 6 Department of Pathology and Immunology, Medical School, University of Geneva, Geneva, Geneva, Switzerland; 7 Department of Radiation Oncology, Mayo Clinic, Rochester, MN USA

**Keywords:** CNS cancer, Cancer stem cells

## Abstract

Recently, we involved the carbohydrate-binding protein Galectin-3 (Gal-3) as a druggable target for KRAS-mutant-addicted lung and pancreatic cancers. Here, using glioblastoma patient-derived stem cells (GSCs), we identify and characterize a subset of Gal-3^high^ glioblastoma (GBM) tumors mainly within the mesenchymal subtype that are addicted to Gal-3-mediated macropinocytosis. Using both genetic and pharmacologic inhibition of Gal-3, we showed a significant decrease of GSC macropinocytosis activity, cell survival and invasion, in vitro and in vivo. Mechanistically, we demonstrate that Gal-3 binds to RAB10, a member of the RAS superfamily of small GTPases, and β1 integrin, which are both required for macropinocytosis activity and cell survival. Finally, by defining a Gal-3/macropinocytosis molecular signature, we could predict sensitivity to this dependency pathway and provide proof-of-principle for innovative therapeutic strategies to exploit this Achilles’ heel for a significant and unique subset of GBM patients.

## Abstract (web summary)

Seguin et al demonstrated in glioblastoma patient-derived stem cells that a subset of glioblastoma tumours is dependent on macropinocytosis mediated survival through a Galectin-3/RAB10/beta 1 integrin axis. They used both genetic and pharmacologic inhibition of Galectin-3 in vivo and in vitro to identify underlying mechanisms and define a Galectin-3/macropinocytosis molecular signature, which could inform the development of anti-tumour therapeutic strategies.

## Introduction

The World Health Organization (WHO) classifies astrocytomas as low grade (grade I to II) or high grade (grade III and IV)^1^. Glioblastoma (GBM) is a grade IV astrocytoma, a deadly malignant brain tumor and the most common primary brain tumor in adults. Today, surgery, radiotherapy, and chemotherapy with temozolomide (TMZ) remain the standard of care in patients with GBM^2^. However, the overall survival of patients with GBM (~14 months) has not radically changed over the past 15 years.

Major efforts in large-scale genomic and transcriptomic profiling allow the characterization and stratification of GBM patients into three major subtypes: proneural, classical, and mesenchymal^3–5^. However, this big data analysis has yet to highlight new avenues and druggable molecules to achieve advances in precision medicine. As shown by three recent precision medicine studies, molecular profiling is not the only route to guide therapy in patients. By using circulating tumor DNA (TARGET study), drug combinations (i-PREDICT study), or DNA sequencing (WINTHER study), these studies took into account the individuals in a more global perspective^6–8^ and clarified how tumors within the same subgroup could differentially respond to therapies.

GBMs are composed of multiple cell types, including cancer stem cells, namely GBM stem cells (GSCs)^9^. These cells display many properties such as self-renewing and tumor-initiating properties, differentiation capacities and they are able to survive the harsh hypoxic and nutrient deprived brain tumor microenvironment. Understanding how they can thrive in these challenging conditions would therefore provide an opportunity to target the most aggressive and drug-resistant cell within the tumor.

Gal-3 belongs to a family of carbohydrate-binding proteins that have a high affinity for β-galactoside-containing glyco-conjugates and have carbohydrate recognition domains that are evolutionary conserved^10^. Three groups have been described: prototype, chimera, and tandem repeat groups, and Gal-3 is the sole representative of the chimera type of the galectin family. Gal-3 is highly expressed in cancer cells and has a broad range of functions related to cell survival, proliferation, invasion, or apoptosis due to its interaction with intra- and extracellular proteins^11^. In cancers, Gal-3 is associated with RAS signaling, and thanks to its carbohydrate recognition domain, Gal-3 can interact with KRAS-GTP, stabilizing it in its active state^12,13^. Gal-3 is consequently translocated to the plasma membrane where it stimulates phosphoinositide 3-kinase (PI3-K) activation. Through this mechanism, Gal-3 and KRAS regulate key processes in cancer cells. Recently, we showed that integrin αvβ3-positive pancreatic and lung cancer cells are uniquely addicted to mutant KRAS and we defined Gal-3 as a critical mediator of this activity, functioning through the regulation of macropinocytosis^14^. We showed how the biochemical association between αvβ3/Gal-3/KRAS can be disrupted by using GCS-100, a specific inhibitor of Gal-3. Indeed, macropinocytosis inhibition was achieved with GCS-100. In addition, there was an increase of ROS levels in KRAS-mutant cells in 3D culture, as well as in tumor xenografts and in PDX tumors^14^.

Macropinocytosis represents an endocytic process that provides cancer cells with the ability to uptake proteins and extracellular fluids into large intracellular vesicles known as macropinosomes^15^. Macropinosomes are large membrane vesicles that form upon the extension and folding of large membrane ruffles back onto the cell surface, thereby mediating the bulk intake of not only extracellular fluids but also proteins^16^. In addition to their large size and the ability to internalize high molecular weight dextran, macropinosomes are also defined by their unique sensitivity to amiloride and its derivatives such as 5-(Nethyl-N-isopropyl) amiloride (EIPA), which represent the most effective and selective agent currently used to pharmacologically inhibit macropinocytosis^17^. In the harsh tumor microenvironment, where no/low nutrients and oxygen are available, macropinocytosis can represent a unique mechanism of amino acid uptake to allow survival. In cancer, macropinocytosis can be driven not only by oncogenes, such as RAS and SRC, but can also be stimulated by growth factors (e.g., EGF), and/or ruffling kinase (e.g., p21-activated kinase-1) to modulate cancer cell metabolism and nutrient internalization^14,16^. Commisso and colleagues have revealed oncogenic KRAS-mediated macropinocytosis as an entry route for extracellular albumin in 2D pancreatic cancer cell lines. In this context, macropinocytosis represents a critical nutrient delivery pathway that cancer cells use to allow their survival in the challenging tumor microenvironment.

In the macropinocytic pathway, several RAB (for Ras-related protein in the brain) proteins have been shown to be involved in macropinosome formation and subsequent macropinosome maturation^18^. RAB5 represents the best-characterized member of this family for macropinosome formation^19^. Several studies have already described how RAB5 is recruited to ruffles when phosphatidylinositol 3,4,5-triphosphate (PI(3,4,5)P3) is generated at the plasma membrane^20^. In RAW264 macrophages, Egami et al.^21^ showed that RAB21 and RAB20 (which are close homologs of RAB5) are recruited to RAB7-positive maturing macropinosomes.

Here, we report that Gal-3-mediated macropinocytosis allows survival of a subset of GBM cells. By identifying and characterizing a subpopulation of patient-derived Gal-3^high^ GSCs sensitive to Gal-3 and macropinocytosis blockade, we describe a non-oncogenic (KRAS) cellular context with enhanced macropinocytosis activity. Indeed, no KRAS mutations have been reported in GBM^3,4^. Remarkably, we demonstrate that Gal-3 can bind to RAB10, which is required for macropinocytosis. However, no studies have reported the role of RAB10 in the macropinocytosis process, whereas its function in late endosome formation is well documented. Finally, we define a Gal-3/macropinocytosis molecular signature that can be used to predict sensitivity to this dependency pathway. By identifying this dependency pathway defined by a molecular signature rather than an oncogenetic status, we provide proof-of-principle for new therapeutic strategies to exploit this vulnerability for a significant subset of GBM patients and potentially for other WT KRAS cancers showing macropinocytosis addiction.

## Results

### High Gal-3 mRNA expression correlates with poor survival and is associated with macropinocytosis in mesenchymal subtype GBM

**Fig. 1:**
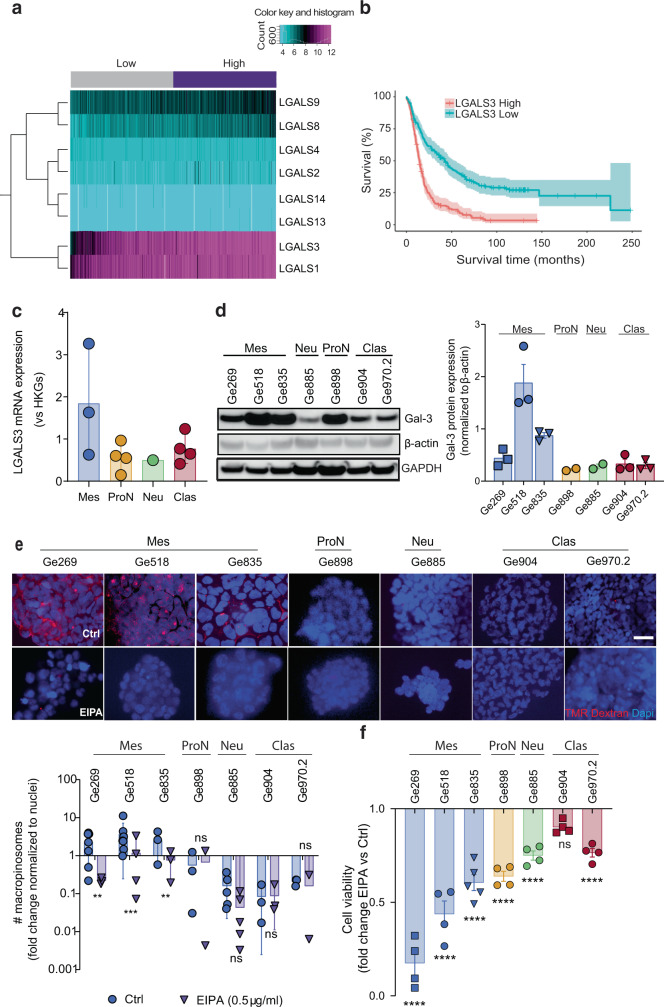
Gal-3 levels correlate with poor survival and macropinocytosis rate in GBM. **a** Hierarchical clustering of galectin-3 expression correlated to a risk score predicting patient survival for the TCGA GBM dataset (*n* = 538 patients). Low = low-risk group; high: high-risk group. **b** Kaplan–Meier analysis of Rembrandt dataset for Gal-3 expression (*n* = 179 Gal-3^low^, *n* = 136 Gal-3^high^; *p* < 0.0001). **c** Gal-3 mRNA expression was determined by qPCR in GSCs. HKGs = housekeeping genes. **d** Immunoblots showing the expression of Gal-3 in GSCs. The histogram represents Gal-3 normalized to loading control (β-actin) determined by densitometry analysis. **e** Macropinocytosis uptake assay using TMR-dextran as a marker of macropinosomes (in red) in GSCs under EIPA or not. Scale bar, 10 µm. Histograms represent the fold change of macropinocytosis activity in all GSCs normalized to nuclei number (*n* = 2–5). **f** Effect of EIPA on cell viability measured by CellTiter-Glo in GSCs (*n* = 4–5). Data are represented as mean ± SEM (*p < 0.05, **p < 0.01 and ***p < 0.001), two-way ANOVA, Sidak’s adjusted *p* value. ns nonsignificant, Ctrl Vehicle (DMSO), Mes mesenchymal, ProN proneural, Neu neural, Clas classical.

Recently, we found that GSC with a mesenchymal signature showed a highly glycolytic expression signature, which is not correlated with a dependence on the high-affinity glucose transporter type III, GLUT3, for their survival. Indeed, GLUT3 is the gatekeeper of the glycolytic pathway and a known driver of a cancer stem cell phenotype. Because our previous study showed that Gal-3 gives rise to mutant KRAS addiction by directly binding to the cell surface receptor integrin αvβ3, we hypothesized that Gal-3 could mediate macropinocytosis allowing mesenchymal GSC to survive in the stressful brain tumor microenvironment. We first considered whether Gal-3 (*LGALS3*) expression has clinical relevance in GBM. To do so, we examined the correlation between its expression and patient survival in several GBM databases. Our analysis revealed Gal-3 is the only galectin whose mRNA expression consistently correlates with poor survival in several datasets (Fig. 1A and Supplementary Fig. 1A and Supplementary Table 1). Moreover, we showed that Gal-3 expression increases along with astrocytoma grade and is highly expressed by pseudopalisading cells, and by GSCs with a mesenchymal signature (Supplementary Fig. 1B, C and Fig. 1B). In accordance, for our modest cohort of GBM biopsies, we found that all GBM specimens showed expression of Gal-3 by immunohistochemistry with a trend towards a higher expression for GBM enriched for mesenchymal genes (Supplementary Fig. 1D). Then, to evaluate how high Gal-3 expression could lead to poor survival in GBM, we performed a differential gene expression analysis based on Gal-3^high^ versus Gal-3^low^ in GBM patients in several datasets (Supplementary Table 2). The gene ontology enrichment analysis revealed expression of genes involved in an extracellular matrix organization (*CD44*, *LAMB1*, *TNC*, *TIMP1*, *LOX*, *COL1A2*, *SERPINE1*), angiogenesis (*VEGFA*, *TGFβI*, *COL4A2*), endocytosis (*CAV1*, *ANXA1*, *ANXA2*, *SH3GL2*), and collagen catabolism/metabolism (*COL3A1*, *COL5A2*, *LUM*), which are in line with previous studies (Supplementary Fig. 1D). Moreover, for a large majority of these genes, their expression is correlated with poor survival in GBM (Supplementary Fig. 2A–C).

By analyzing bulk tumors with image-guided multiregional sampling, transcriptomic profiling has been associated with specific tumor regions/microenvironments^22^. Therefore, a mesenchymal transcriptomic signature has been associated with a necrotic area and pseudopalisading cells (surrounding these necrotic areas), which are defined by their nutrient-deprived and hypoxic microenvironment. In such a harsh microenvironment, cancer cells need to overcome challenges imposed by this deprivation. We reasoned that GSCs with a mesenchymal signature might overcome the necrotic microenvironment by inducing Gal-3 mediated macropinocytosis^14^. In accordance with this hypothesis, we observed a positive correlation between Gal-3 expressing GSCs with a mesenchymal signature and a higher rate of macropinocytosis visualized by TMR-dextran uptake (Supplementary Fig. 2D and Fig. 1C–E. Consistent with these findings, treatment with EIPA, an inhibitor of macropinocytosis that does not affect other endocytic pathways, induced a greater decrease of macropinocytosis-mediated cell viability in mesenchymal GSCs as measured by the quantification of metabolically active cells (Fig. 1E). Of note, chlorpromazine, an inhibitor of clathrin-mediated endocytosis, did not affect GSC survival (Supplementary Fig. 2E). Finally, GSC invasive capacity was completely blocked upon EIPA treatment only in mesenchymal GSCs, indicating that macropinocytosis was required only for mesenchymal GSC invasion (Supplementary Fig. 2F). By using a transcriptomic signature generated from a list of the top 25 Gal-3/survival-associated genes predicted to identify the addicted *vs.* non-addicted phenotype (Supplementary Table 2), we were able to split our GSCs according to Gal3-/macropinocytosis addicted *vs.* non-addicted (Supplementary Fig. 2G).

### Gal-3 is required for macropinocytosis-mediated mesenchymal GSCs survival and invasion in 3D

**Fig. 2:**
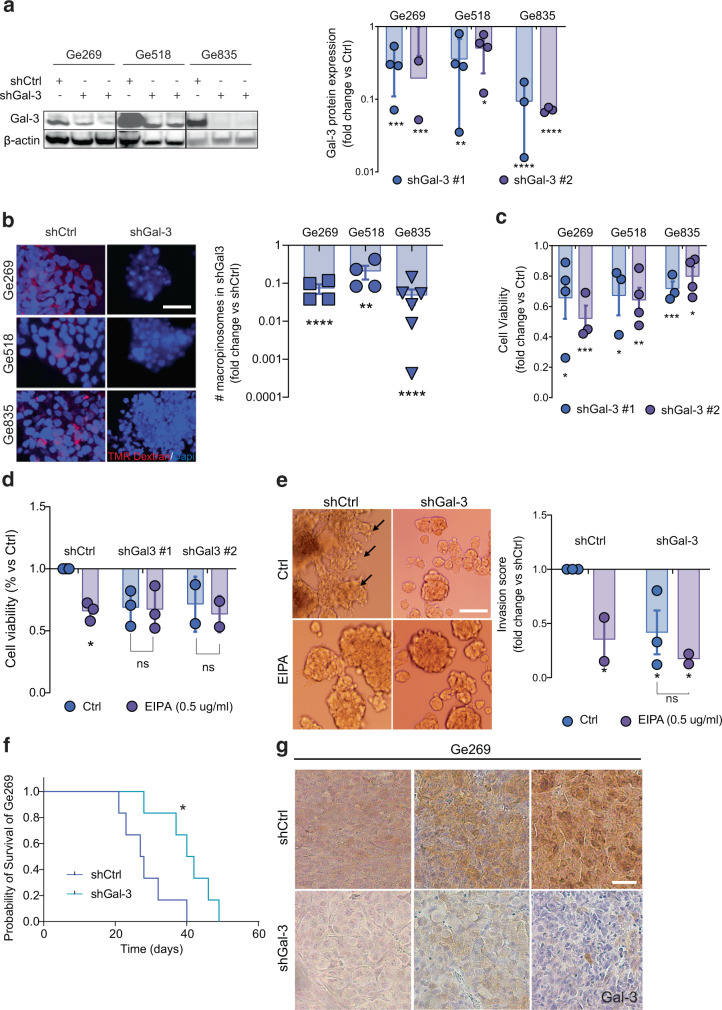
Gal-3 is required for macropinocytosis. **a** Immunoblots show expression of indicated proteins for Ge518, Ge269, and Ge835 infected by shRNA Control (Ctrl) or shGal-3. Histograms show the fold change of protein expression determined by densitometry analysis. **b** Macropinocytosis uptake assay using TMR-dextran in shCtrl *vs.* shGal-3 #2 GSCs. Scale bar, 10 µm. Histograms represent the fold change of macropinocytosis activity in Ge518, Ge269, and Ge835 normalized to nuclei number (*n* = 4–6). Ctrl = Vehicle (DMSO). **c** Cell viability of Ge518, Ge269, and Ge835 in shRNA Control (Ctrl) *vs.* shGal-3, measured by CellTiter-Glo in GSCs (*n* = 3–4). **d** Cell viability of Ge518 shRNA Control (Ctrl) *vs.* shGal-3 under EIPA treatment, measured by CellTiter-Glo in Ge518 (*n* = 2–3). Data are represented as mean ± SEM (*p < 0.05, **p < 0.01, and ***p < 0.001), two-way ANOVA, Dunnett’s multiple comparisons test. **e** Cell invasion in 3D of Ge518 shRNA Control (Ctrl) *vs.* shGal-3 under EIPA treatment (*n* = 2–3). Scale bar, 100 µm. Histograms represent the fold change of the invasion score in Ge518. Data are represented as mean ± SEM (*p < 0.05, **p < 0.01, and ***p < 0.001), two-way ANOVA, Dunnett’s multiple comparisons test. **f** Effect of Gal-3 knockdown on tumor growth in vivo: Ge269 shCtrl *vs.* shGal-3, (*n* = 7 mice per group), *p* = 0.016 (Log-rank Mantel–Cox test). **g** Histological analysis of Ge269 shCtrl *vs.* shGal-3. Tumors were stained for Gal-3, and counterstained with hematoxylin. (*n* = at least 3 mice per group). Scale bar, 50 µm. Data are represented as mean ± SEM (*p < 0.05, **p < 0.01, and ***p < 0.001), two-way ANOVA, Sidak’s adjusted *p* value. ns = nonsignificant, Ctrl = Vehicle (DMSO).

In GBM, Gal-3 has been described for its critical role in cancer survival and invasion. However, Gal-3 implication in macropinocytosis-mediated survival or invasion has not been addressed before in the context of GBM, in which oncogenic KRAS is not present. To investigate whether Gal-3 expression is required for macropinocytosis, we knocked down Gal-3 in mesenchymal GSCs, which express high levels of Gal-3 (Fig. 2A and Supplementary Fig. 2E). Remarkably, our results showed that shRNA-mediated Gal-3 knockdown strongly inhibits macropinocytosis in mesenchymal GSCs Ge269, Ge518, and Ge835 as shown by lower dextran uptake (Fig. 2B). Functionally, we showed that either macropinocytosis or Gal-3 inhibition induced a decrease in cell viability in mesenchymal GSCs (Fig. 2C). As EIPA treatment does not affect the viability of Gal-3 knockdown cells, our data indicate that Gal-3 could largely account for the regulation of macropinocytosis (Fig. 2D). Remarkably, we showed that only the subset of tumors expressing high Gal-3 are dependent on Gal-3 for survival. Indeed, Gal-3 knockdown in GSCs Ge904 or Ge885 (Gal-3^low^), did not, or barely affect, their survival (Supplementary Fig. 3A, B). Moreover, we observed an inhibition of cell invasion in Gal-3 knockdown mesenchymal GSC compared to their control (Fig. 2E). Importantly, knockdown of Gal-3 significantly delayed the orthotopic growth of GBM tumors with a mesenchymal signature in nude mice, indicating that Gal-3 promotes GSC survival and tumorigenic capacity in the brain (Fig. 2F, G). Moreover, we observed a trend towards a decrease of tumor cell invasion and dissemination into the adjacent healthy parenchyma as well as a decrease of tumor vascularization in shGal-3 tumors (Supplementary Fig. 3C, D). Collectively, we show that Gal-3 is required for macropinocytosis-mediated mesenchymal GSC survival, invasion, and tumorigenic capacity.

### Gal-3 expression is modulated by hypoxia and activates Akt signaling

**Fig. 3:**
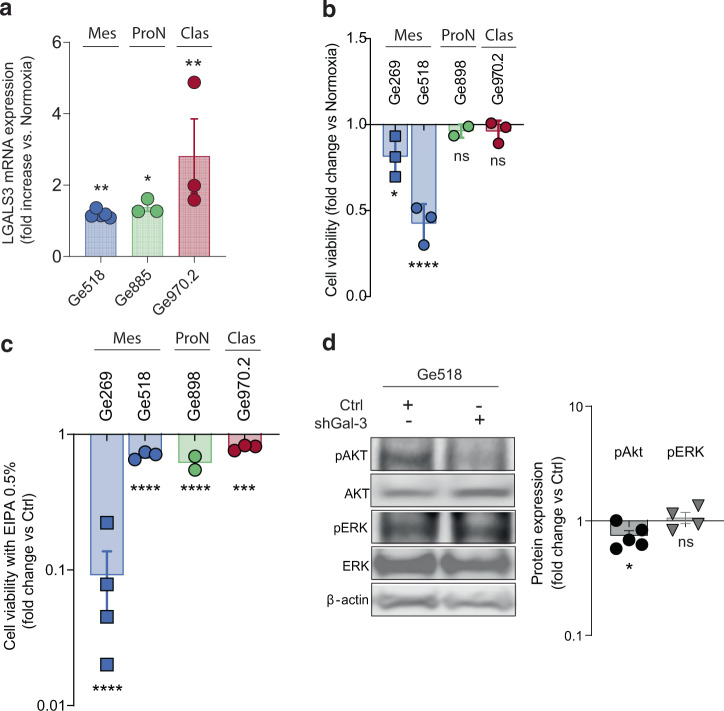
Gal-3 expression is modulated by hypoxia and activates Akt. **a** Effect of hypoxia (1%) during 48 h on Gal-3 expression determined by qPCR in GSCs and normalized to housekeeping genes (HKGs) (*n* = 3–5). **b** Effect of hypoxia (1%) on cell viability during 72 h, measured by CellTiter-Glo. **c** Effect of EIPA on cell viability under hypoxic conditions during 72 h, measured by using CellTiter-Glo (*n* = 2–4). **d** Immunoblots showing expression of indicated proteins when Gal-3 is knocked down in Ge518. Histograms represent the fold change of protein expression determined by densitometry analysis (*n* = 4). Data are represented as mean ± SEM (*p < 0.05, **p < 0.01, and ***p < 0.001), two-way ANOVA, Sidak’s adjusted *p* value. ns nonsignificant, Mes mesenchymal, ProN proneural, Clas classical, Neu neural.

Several studies have shown that Gal-3 expression is part of an adaptive response that protects GBM cells from death under hypoxia, explaining why mesenchymal GSCs, found in the hypoxic and necrotic areas, express a higher level of Gal-3 compared to other GSC subtypes^23,24^. To test whether GSCs could modulate Gal-3 expression under hypoxic conditions regardless of their subtype, we exposed some of our GSC to 1% oxygen then monitored Gal-3 expression as well as cell viability. As expected, Gal-3 expression was induced upon exposure to hypoxia (Fig. 3A). Nevertheless, we observed a decrease of viability under hypoxia only for the mesenchymal GSCs, suggesting that mesenchymal GSCs might be more sensitive to hypoxic conditions (Fig. 3B). However, we did not observe a consistent response of cell viability to EIPA under hypoxic conditions, suggesting a general cellular response to stress (Fig. 3C). Consistent with previous studies, we also observed a decrease of AKT activation upon Gal-3 knockdown in mesenchymal GSCs whereas we could not observe any modulation of AKT activation upon Gal-3 knockdown in Ge885 (Fig. 3D and Supplementary Fig. 3E). Altogether, our data showed that Gal-3 expression is modulated by hypoxia and its downregulation induced a decrease in AKT activation.

### Inhibition of Gal-3 reveals its downstream molecular targets

**Fig. 4:**
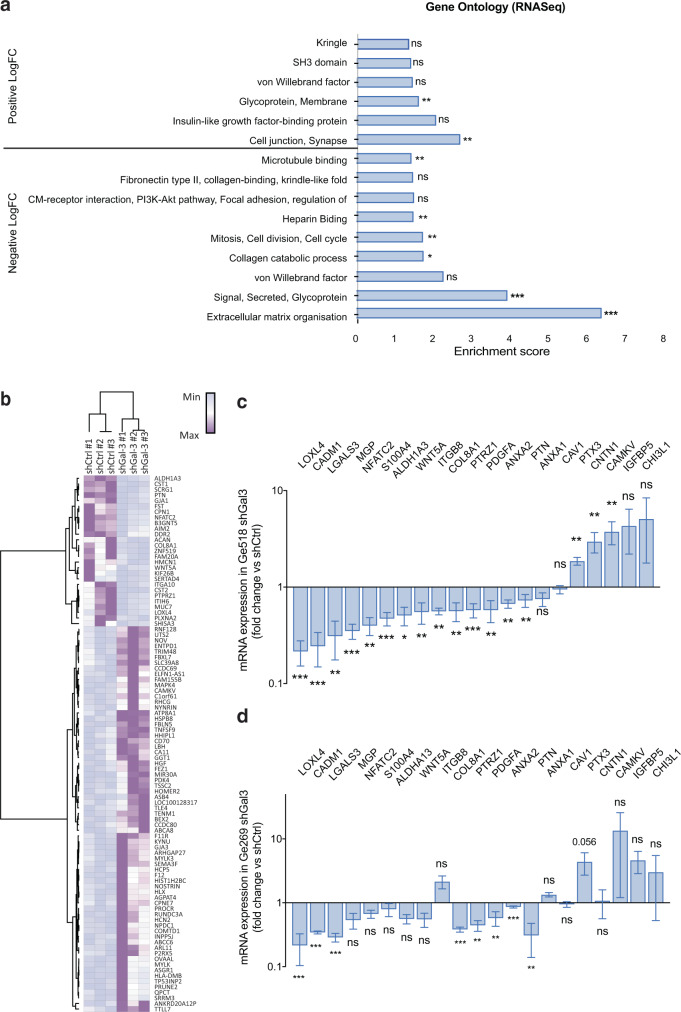
PTPRZ1, PDGFa, ANXA2, and COL8A1 expression are modulated by Gal-3. **a** Functional annotation clustering of gene set enrichment analysis comparing Ge518 shCtrl *vs.* shGal-3. Histograms show the enrichment score of each family of genes. **b** Hierarchical clustering of Ge518 shCtrl *vs.* shGal-3 based on the differential expressed genes. **c** mRNA expression was determined by qPCR in Ge518 infected by shRNA Control (shCtrl) *vs.* shGal-3 (*n* = 2–3). **d** mRNA expression was determined by qPCR in Ge269 infected by shCtrl *vs.* shGal-3 (*n* = 2–3). Data are represented as mean ± SEM (*p < 0.05, **p < 0.01, and ***p < 0.001), Student *t* test. ns nonsignificant.

The molecular mechanisms regulated by Gal-3 which contribute to its effects in GBM, especially in the context of macropinocytosis, have not yet been described. To do so, we undertook two different strategies: a transcriptomic and a proteomic approach. First, in order to identify Gal-3 downstream effectors, we performed RNASeq analysis of the mesenchymal GSC, Ge518, transduced by shRNA-mediated Gal-3 knockdown versus shRNA control. Then, we performed a differential gene expression analysis after count normalization. As expected, several families of genes involved in ECM–cell signaling, angiogenesis, cell adhesion, and heparin-binding were found (Fig. 4A, B). As shown by previous studies, this analysis suggested that Gal-3 has functional roles in remodeling the tumor microenvironment, cell signaling, collagen catabolism/biogenesis, and angiogenesis. To validate our RNASeq data, we evaluated gene expression by quantitative PCR in Ge518 and Ge269 shGal-3 cells compared to their control and observed a significant modulation of their expression (Fig. 4C, D). Upregulation of several of these genes, for instance, *PTPRZ1*, *WNT5A*, *S100A4*, or *ANXA1/2*, has been linked to cancer invasion and aggressiveness in some solid tumors, including GBM^25–30^. Moreover, their expression is correlated with poor survival in several GBM datasets and, as for Gal-3, enriched in necrotic areas (Supplementary Fig. 3F–H; and Supplementary Table 3). Consequently, by modulating these genes, Gal-3 knockdown could lead to the inhibition of GBM aggressiveness.

### RAB10 interacts with Gal-3 to regulate macropinocytosis

**Fig. 5:**
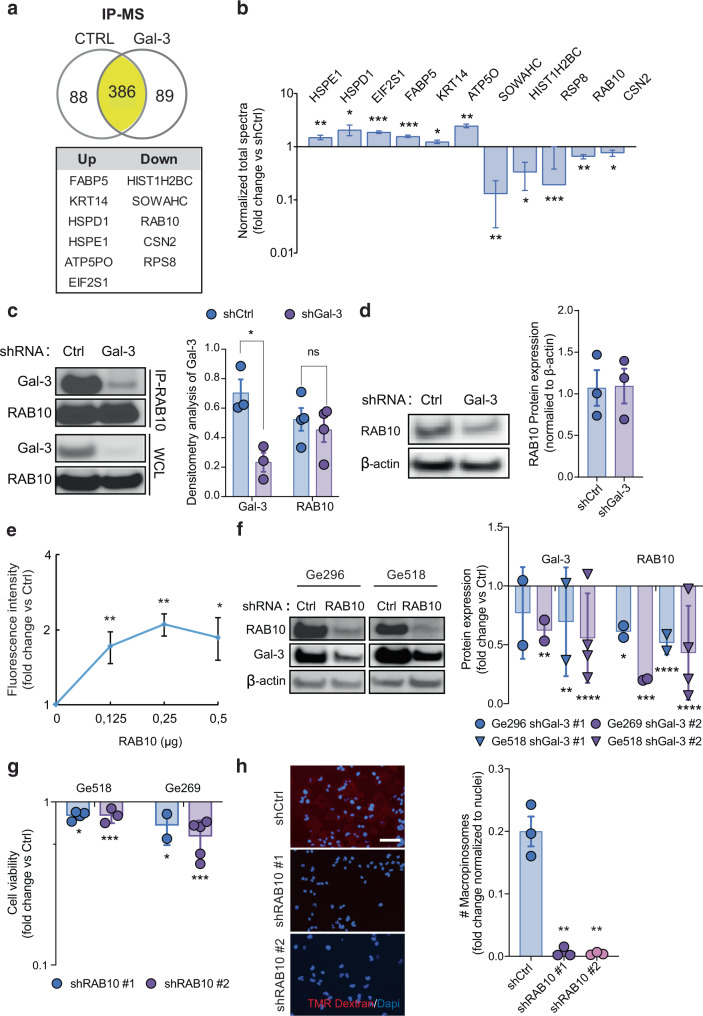
Gal-3/RAB10 interaction is a surrogate for macropinocytosis-mediated GSC survival. **a** The scheme summarizes the IP-MS hits in Ge518 shCtrl *vs.* shGal-3. **b** Histograms represent the fold change of normalized total spectra for significantly identified proteins by IP-MS analysis, in Ge518 shCtrl *vs.* shGal-3. **c** Immunoblot analysis of RAB10 immunoprecipitation from Ge518 shCtrl *vs.* shGal-3. Histograms represent the fold change of Gal-3 and RAB10 expression determined by densitometry analysis (*n* = 3–4). WCL whole-cell lysate. **d** Immunoblots show expression of indicated proteins for Ge518 shCtrl or shGal-3. Histograms show the fold change of protein expression determined by densitometry analysis (*n* = 3). **e** A cell-free binding assay shows direct binding between RAB10 and Gal-3 (*n* = 4). **f** Immunoblots show expression of indicated proteins for Ge518 and Ge269 shCtrl *vs.* shRAB10. Histograms show the fold change of protein expression determined by densitometry analysis. **g** Effect of RAB10 knockdown on cell viability measured by CellTiter-Glo in Ge518 and Ge269. **h** Macropinocytosis uptake assay using TMR-dextran in Ge518 shCtrl *vs.* shRAB10. The histogram represents the fold change of macropinocytosis activity in Ge518 normalized to nuclei number (*n* = 3). Data are represented as mean ± SEM (*p < 0.05, **p < 0.01, and ***p < 0.001), two-way ANOVA, Dunnett’s multiple comparisons test. ns nonsignificant.

As a second strategy, we choose a proteomic approach. Since Gal-3 can interact and complex with multiple molecules, we postulated that immunoprecipitation of Gal-3 combined with liquid chromatography–mass spectrometry (LC–MS) would allow identification of its partner(s) in our cellular context. In total, we detected 563 proteins: 88 proteins were identified only in the control group and 89 proteins were found in the shGal-3 group (Fig. 5A). This approach enabled the identification of eleven Gal-3-binding proteins that are significantly modulated in Ge518 shGal-3 GSC compared to their control (Fig. 5A, B). In shGal-3 GSC, we found Gal-3 significantly associated with FABP5, KRT14, EIF2S1, HSPD1, HSPE1, and ATP5PO. Of great interest, in shCtrl GSC, HIST1H2BC, SOWAHC, RAB10, CSN2, and RPS8 were found significantly associated with Gal-3 (Fig. 5B). Moreover, in shGal-3 GSC, the enrichment analysis revealed the expression of proteins involved in stress response, heat shock protein family, and mitochondrion. In contrast, in shCtrl, we found enrichment for proteins involved in cell adhesion, actin-binding, myosin complex, and translational initiation (Supplementary Fig. 4A).

Because it belongs to the RAS superfamily of small GTPases, which regulate many cellular systems, including membrane trafficking and endocytosis, RAB10 appears as the most interesting hit^31^. Considering that no KRAS mutation is found in GBM, we hypothesized that RAB10 is required for macropinocytosis in GSC with a mesenchymal signature. To confirm our hypothesis, we first performed immunoprecipitation of RAB10 followed by immunoblotting for Gal-3. Accordingly, Gal-3 was found co-immunoprecipitated with RAB10 (Fig. 5C). Of note, we did not find a significant decrease of RAB10 expression in shGal-3 GSCs compared to their control even if we observed a trend toward a lower expression in the knockdown cells (Fig. 5D).

According to Wan et al.^32^, RAB10 was found co-fractionated with Gal-1. In line with these results, Gal-3 can bind to several members of the RAB10 superfamily such as RAB7A (co-localization by IF) and RAB11B (affinity capture-MS) (Supplementary Table 4)^33,34^. Regarding RAB family homology, as well as galectin family homology, we postulated that Gal-3 can directly bind to RAB10. To test our hypothesis, we performed a cell-free binding assay and we revealed that Gal-3 can bind to RAB10 in a saturable manner (Fig. 5E). Moreover, we observed a colocalization between RAB10 and Gal-3 in the mesenchymal GSC Ge518 as already shown by immunoprecipitation (Supplementary Fig. 4B). In this context, cell fractionation also showed enrichment for RAB10 and Gal-3 at the plasma membrane compared to the nuclear and cytosolic compartments in Ge518 GSC (Supplementary Fig. 4C). However, we observed no effect of Gal-3 on RAB10 loading with the BODIPY-GTP, indicating that Gal-3 may only serve as an anchor for RAB10 at the plasma membrane (Supplementary Fig. 4D). Collectively, our results showed that Gal-3 could physically interact with RAB10 at the plasma membrane.

Because several RAB proteins have been described to be required or involved in macropinosome formation/maturation, we postulated that RAB10 mediates this function in mesenchymal GSCs. Furthermore, we first knocked it down in Ge518 and Ge269 mesenchymal GSCs. However, no GSCs survived after shRAB10 transduction. RAB GTPases family represents a master regulator of the secretory and endocytic pathways and guarantees membrane integrity^20^. More importantly, the targeted disruption of RAB10 leads to early embryonic lethality^35^. Therefore, it is reasonable to think that RAB10 knockdown might be toxic to GSCs due to its strong impact on key cellular processes. Because cancer cells rely on different molecules that mediate survival when they are adherent, we decided to generate 2D Ge518 and Ge269 cell lines derived from our GSCs (named GDC for GBM differentiated cells). In accordance with our hypothesis, cell viability was reduced after RAB10 knockdown in Ge518 and Ge269 GDC models, (Fig. 5F, G). Moreover, we found a decrease of TMR-Dextran uptake in GDC Ge518 transduced by shRNA-mediated RAB10 knockdown (Fig. 5H). In addition, as for Gal-3 expression, RAB10 expression was correlated with Gal-3 and was shown to be enriched in pseudopalisading GBM cells (Supplementary Fig. 5A, B). Unlike Gal-3, we found a high expression of RAB10 in all our GBM biopsies, and, furthermore, its expression did not consistently correlate with patient survival (Supplementary Fig. 5C–E). More interestingly, as for Gal-3 expression, the gene ontology enrichment analysis based on RAB10^high^ *vs.* RAB10^low^ in GBM patients in the Rembrandt dataset revealed expression of genes involved in several processes, such as regulation of vesicle-mediated transport, receptor-mediated endocytosis, neurotransmitter transport or chloride transport (Supplementary Fig. 5F). Focusing on the receptor-mediated endocytosis family of genes found in both gene ontology enrichment analysis for Gal-3 and RAB10, we also identified ANXA2, SERPINE1, VEGFA, SH3GL2, SNAP91, and CAV1, overlapping the reverse-phase protein array analysis from TCGA (Supplementary Table 5). However, we could not find significant differences for ANXA1/ANXA2, ITGB3/ITGB1 between shGal-3 GSCs and their control (Supplementary Fig. 6A). Of note, even if we could not achieve significance due to the absence of ITGB1 or ICAM in one sample pair, we could observe a trend towards a decrease of expression. Finally, as for Gal-3 knockdown, we observed a decrease of AKT activation upon RAB10 knockdown in Ge518, suggesting that RAB10 may have a broader impact on GSC downstream signaling (Supplementary Fig. 6C). Altogether, our data identified RAB10 as a partner of Gal-3 required for macropinocytosis in GSCs. To investigate whether ectopic expression of Gal-3 and RAB10 is sufficient to drive macropinocytosis in a non-mesenchymal GSC, we transfected the pEGFP-Gal-3 and transduced the PLX-307-RAB10 plasmids in Ge904 (Supplementary Fig. 4E). As shown by an increase of TMR-Dextran uptake, ectopic expression of Gal-3 and RAB10 lead to an enhanced macropinocytosis activity in Ge904 expressing high Gal-3 and RAB10 (Supplementary Fig. 4F).

We have previously shown that Gal-3 gives rise to KRAS addiction by directly binding to the cell surface receptor integrin αvβ3 in non-small cell lung cancer and pancreatic carcinoma cells^14^. To provide mechanistic insight into Gal-3 and RAB10 regulation of macropinocytosis, we performed an additional immunoprecipitation of Gal-3 combined with LC-MS in Ge269 shGal-3 and shCtrl. When we compared both LC–MS analyses from Ge518 and Ge269, RAB10, and β1 integrin were both found associated with Gal-3 (Supplementary Fig. 6A, B). Then, based on our results and the literature, we hypothesized that β1 integrin forms a cluster with Gal-3 and RAB10 to mediate macropinocytosis. Of note, even if *ITGB1* expression was not consistently found correlated with GBM patient survival, its expression was significantly found associated with the mesenchymal subtypes in several GBM datasets^36^. As shown by β1 integrin and Gal-3 co-immunoprecipitation, our results showed an interaction between both proteins (Supplementary Fig. 7A). The pharmacologic inhibition of β1 integrin with a blocking antibody, P4C10, induced a decrease of cell viability in the mesenchymal GSC Ge269 and Ge518 but not in the non-mesenchymal GSC Ge885, Ge904, and 970.2 (Supplementary Fig. 7B). In line with previous studies showing that Gal-3 interacts with β1 integrin^37^, we also observed a colocalization between Gal-3 and β1 integrin in the mesenchymal GSC Ge518 which is not the case for Ge885 where Gal-3 was found in the cytosol (Supplementary Fig. 7C). Finally, the genetic or pharmacologic knockdown of β1 integrin leads to a significant inhibition of macropinocytosis visualized by TMR dextran uptake (Supplementary Fig. 7D). Mechanistically, our results showed that the Gal-3/RAB10/β1 integrin cluster is required to regulate macropinocytosis in mesenchymal GSCs.

### A transcriptomic signature can be used to predict sensitivity to Gal-3/macropinocytosis blockade

**Fig. 6:**
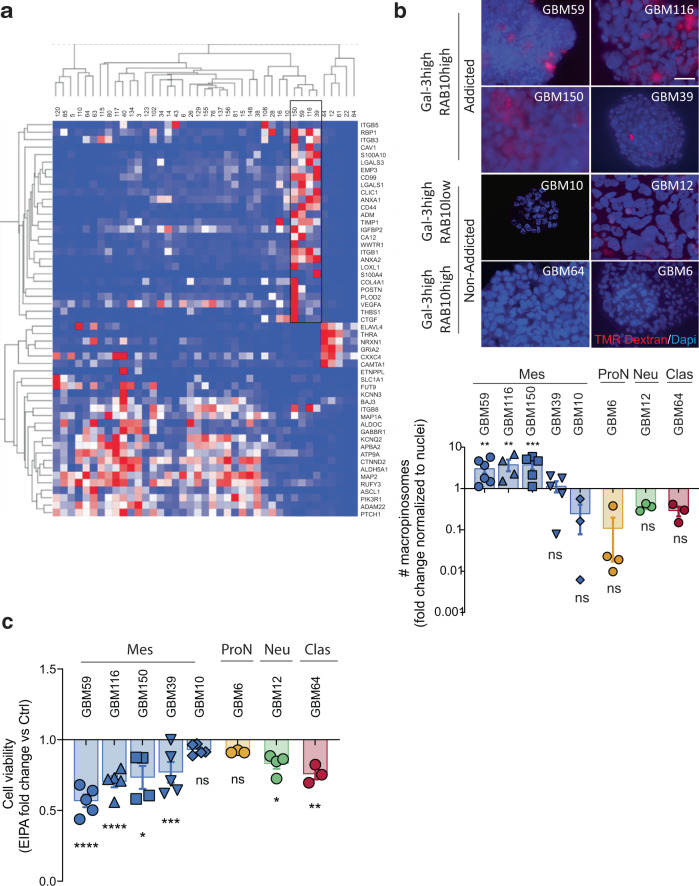
Macropinocytosis blockade can be predicted by a transcriptomic signature. **a** Heatmap showing the transcriptomic signature used for the Mayo Clinic sample request. Samples were requested based on their macropinocytosis addicted *vs.* non-addicted signature. The Black dotted rectangle represents Gal-3/macropinocytosis addicted patients. **b** Macropinocytosis uptake assay using TMR–dextran in Mayo Clinic GSC samples. Histograms represent the fold change of macropinocytosis activity in all GSCs normalized to nuclei number (*n* = 3–6). **c** Effect of EIPA on cell viability measured by CellTiter-Glo in GSCs (*n* = 3–5). Data are represented as mean ± SEM (*p < 0.05, **p < 0.01, and ***p < 0.001), two-way ANOVA, Sidak’s adjusted *p* value. ns nonsignificant, Mes mesenchymal, ProN proneural, Clas classical.

To evaluate how the Gal-3/macropinocytosis addiction status could be predicted, we used the differential gene expression analysis based on Gal-3^high^ *vs.* Gal-3^low^ generated from the TCGA dataset (Supplementary Table 2). Subsequently, we generated a list of Gal-3/survival-associated genes predicted to identify and to confirm the strength of our signature to predict sensitivity to Gal-3/macropinocytosis inhibitors. We first interrogated the TCGA dataset to evaluate the subset of macropinocytosis-addicted GBM (Supplementary Fig. 8A). Our data showed that our signature identified about 20% of GBM specimens and it mostly encompassed mesenchymal GBM. However, a few classical and one-proneural GBM were present, indicating that it is not only mesenchymal GBM patients that can be targeted (Supplementary Fig. 8A). As a training cohort, we then requested GSCs from the Mayo Clinic Brain Tumor Patient-Derived Xenograft National Resource based on their expression of genes associated with the Gal-3 addicted *vs.* non-addicted signature (Fig. 6A). Similar to Ge835, Ge518, and Ge269, we observed a higher rate of TMR-dextran uptake for the mesenchymal GSCs GBM59, GBM116, and GBM150, which show high Gal-3 and RAB10 expression (Fig. 6B and Supplementary Fig. 8B, C). Of note, the mesenchymal GSCs GBM39 showed a lower rate of TMR–dextran uptake compared to the other mesenchymal Gal-3-addicted models, and GBM10, with low RAB10 expression, showed no macropinocytosis activity. In contrast, the other classical and proneural GSCs did not (GBM6) or barely showed uptake of TMR-dextran (GBM12 and GBM64), validating the strength of our signature (Fig. 6B). Moreover, we found sensitivity to macropinocytosis inhibitor, EIPA, for all the mesenchymal GSCs GBM59, GBM116, GBM150, and GBM39 (Fig. 6C). In contrast, GBM12, GBM10, and GBM6 were not significantly affected by EIPA treatment. Nevertheless, we did observe a decrease of GBM64 cell viability under EIPA treatment, whereas GBM64 belongs to the classical subtype, probably due the inhibition of sodium-hydrogen exchanger (NHE) activity (Fig. 6C). Of note, as for GBM biopsies, RAB10 was found expressed in all GSCs subtypes (Supplementary Fig. 8D). Remarkably, our transcriptomic signature does not correlate with any consistent mutation profile in our different GSC models (where no common driver mutations were found for GBM59, GBM116, and GBM150) (Supplementary Table 6). Collectively, with our patient cohort in Geneva and our training cohort from the Mayo clinic, we were able to predict sensitivity to macropinocytosis inhibition based only on their transcriptomic signature. Our study emphasizes how precision medicine should take into account not only a transcriptomic signature but rather combined molecular and cellular signatures to guide therapy in GBM patients. More importantly, our results revealed an enhanced macropinocytosis activity regulated by Gal-3 and RAB10 in a non-oncogenic mutant KRAS context for a subset of GBM.

### Galectin-3 inhibitor disrupts Gal-3/RAB10 binding and inhibits macropinocytosis-mediated GSC survival

**Fig. 7:**
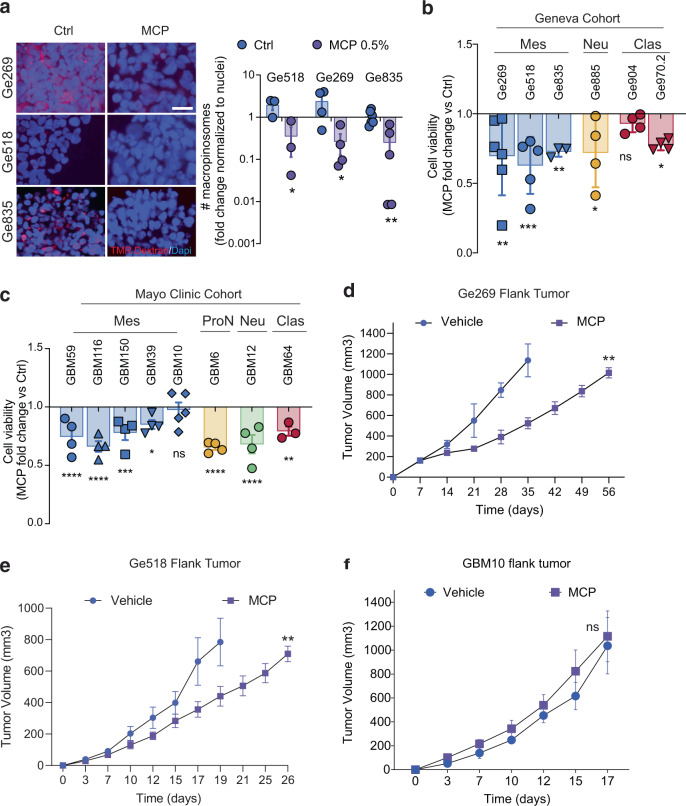
MCP disrupts Gal-3/RAB10 binding and inhibits macropinocytosis-mediated GSC survival. **a** Macropinocytosis uptake assay using TMR-dextran in GSCs. Histograms represent the fold change of macropinocytosis activity in Ge518, Ge269, and Ge835 treated with MCP compared to their control (*n* = 3–5). Scale bar, 10 µm. **b** Effect of MCP on cell viability measured by CellTiter-Glo in GSCs (*n* = 3–6). **c** Effect of MCP on cell viability measured by CellTiter-Glo in GSCs (*n* = 3–5). **d**–**f** Mice were randomized and treated with either vehicle control (*n* = 5) or MCP (*n* = 5; 1% orally in the drinking water) after injection of Ge269, Ge518, and GBM10 cells, respectively. Data are represented as mean ± SEM (*p < 0.05, **p < 0.01, and ***p < 0.001), two-way ANOVA, Sidak’s adjusted *p* value. ns nonsignificant, Ctrl vehicle (H_2_O).

Modified citrus pectin (MCP) antagonizes Gal-3 by binding to the carbohydrate recognition domain of Gal-3, and consequently, impairs Gal-3 functions related to its carbohydrate binding^38–40^. Then, we sought to investigate whether our findings can be translated to the clinic. As a proof-of-concept, we tested whether Gal-3 inhibition with MCP could induce a decrease in macropinocytosis and in macropinocytosis-mediated survival in our GSCs. Consistent with our results, we found a significant inhibition of TMR–dextran uptake in the mesenchymal GSCs, Ge518, Ge269, and Ge835 following MCP treatment (Fig. 7A). Accordingly, GSC survival was significantly affected under MCP treatment in the mesenchymal GSCs but also in some of the non-mesenchymal GSCs (Ge970.2 and Ge885), confirming that MCP effects are not restricted to Gal-3 inhibition (Fig. 7B)^41^. Accordingly, in the Mayo Clinic Training cohort, we also found a significant decrease in GBM6, GBM12, and GBM64 cell viability under MCP treatment (Fig. 7C). Moreover, treatment with MCP of Ge904, ectopically expressing high Gal-3, and RAB10-mediated significant increase of macropinocytosis induced a significant decrease of their viability (Supplementary Fig. 9A).

Moreover, we observed a significant decrease of Gal-3 specific co-immunoprecipitation with RAB10 by performing immunoprecipitation of RAB10 followed by immunoblotting for Gal-3 on Ge518 treated with MCP (Supplementary Fig. 9B). Consistently, we showed that RAB10/Gal-3 interaction can be blocked with MCP by using a cell-free binding assay (Supplementary Fig. 9C). In line with these results, MCP induced a decrease in β1 integrin and Gal-3 colocalization as visualized by immunofluorescence (Supplementary Fig. 9D). In vivo, Ge269 and Ge518 mice bearing tumors orally treated with 1% MCP show a significantly smaller tumor burden (Fig. 7D, E). In contrast, we did not observe any difference in GBM10 mice bearing tumors between the untreated and the MCP treated group (Fig. 7F). Collectively, our results provide a proof-of-concept and a strong rationale for testing Gal-3 inhibition for a subset of patients in GBM therapy.

## Discussion

Macropinocytosis has been described as an endocytic process that allows cancer cells to uptake nutrients (proteins and extracellular fluids). Commisso et al.^16^ have shown that KRAS mutant pancreatic tumors benefit from enhanced macropinocytosis. Recently, we showed that KRAS-dependent lung tumors rely on macropinocytosis driven by the αvβ3/KRAS/Gal-3 complex^14^. Here, we provide evidence that enhanced macropinocytosis-mediated survival can be driven in a WT KRAS context. Here, we report Gal-3/RAB10/β1 integrin interaction as a key modulator of macropinocytosis in a subset of GBM patients. The biochemical association between Gal-3, RAB10, and β1 integrin as well as macropinocytosis inhibition was achieved with MCP, a natural inhibitor of Gal-3. Moreover, our findings highlight macropinocytosis as an important contributor to mesenchymal GSCs survival. Indeed, our data showed that Gal-3^high^ GSCs are uniquely addicted to macropinocytosis and defined a transcriptomic signature that can be used to predict Gal-3/macropinocytosis addiction. Collectively, our findings raise the question of whether macropinocytosis blockade can be envisaged in a subset of WT KRAS cancer cells.

Gal-3 expression has clinical relevance in GBM as its expression is correlated with poor survival. Gal-3 is found highly expressed within pseudopalisading cells that surround the perinecrotic area. Accordingly, GBM cells with a mesenchymal signature are enriched for Gal-3. Indeed, thanks to gene expression profiles (using image-guided multiregional glioblastoma sampling), Jin and et al.^22^ have shown that tumor cells from the perinecrotic region show high expression of mesenchymal genes, confirming data provided by the IvyGAP. To survive and avoid cell death, mesenchymal GBM cells residing in this harsh microenvironment need to use alternative cellular processes to fuel themselves. In our previous study, we showed that mesenchymal GSCs are sensitive to glucose deprivation but are not affected by Glut3 (an upregulated glucose transporter in GBM cells) knockdown, suggesting the existence of alternative pathways^36^. On studying Gal-3 expression in GBM cells exposed to a variety of stress stimuli, Ikemori and colleagues found that NF-κB inhibition by specific proteasomal inhibitors decreased the expression of Gal-3 leading to apoptotic processes^23^. Indeed, Gal-3 expression itself protects GBM cells from apoptosis, and its knockdown induces cell death and delays tumor growth in vivo in U87MG (2D established GBM line). In addition, Gal-3 expression can be modulated by hypoxia and nutrient-deprived conditions as well as by RUNX2 and HIF-1α^24,42^. Our results, in GSCs, remain in line with these previous studies as we found an upregulation of Gal-3 expression under hypoxic conditions. Interestingly, we showed that only a subset of tumors expressing high Gal-3 is dependent on Gal-3 for survival. However, the pharmacologic knockdown of Gal-3 with MCP induces a decrease in cell survival even for non-mesenchymal GSCs. Moreover, considering that all our GSCs express Gal-3, our findings point out the relevance of Gal-3 targeting for a subset of GBM and the importance of precision medicine.

Using a loss-of-function approach, we have determined that Gal-3 is required for macropinocytosis-mediated survival of mesenchymal GSCs. In an attempt to decrypt Gal-3 signaling, we used and compared gene expression profiles between Gal-3^high^ versus Gal-3^low^ samples in GBM patients from different GBM datasets. We showed that Gal-3^high^ co-expressed genes are enriched for a mesenchymal signature. The gene ontology enrichment analysis revealed genes involved in several cellular processes that were consistent with the described functions of Gal-3^24^. Of note, several genes involved in the regulation of endocytosis were identified such as ANXA2, SERPINE1, SH3GL2, SNAP91, and CAV1^28,43^. However, our pull-down assay of Gal-3 combined with LC-MS did not confirm these candidates, but rather allowed the identification of RAB10 and β1 integrin as potential modulators of macropinocytosis in our mesenchymal GSC models. By interacting with Gal-3, KRAS-GTP enhances the translocation of Gal-3 to the plasma membrane, thereby promoting PI3-K/Akt downstream signaling to stimulate macropinocytosis^44^. As for KRAS, active RAB members carry GTP and are associated with the plasma membrane^45^. One of the most interesting outcomes of our study is the identification of RAB10 not only as a direct binding partner of Gal-3 but also as a modulator of macropinocytosis in mesenchymal GSCs. Several studies have already investigated how different RABs (RAB5, RAB7, RAB20, and RAB21) are recruited to ruffles and are involved in macropinocytosis^18^. Our data reveals that the interaction between Gal-3 and RAB10 represents a critical contextual signal that is required for macropinocytosis. As such, we believe that macropinocytosis can occur in a subset of GBM cells that highly express Gal-3 and RAB10. Consistent with this idea, we did not observe enhanced macropinocytosis in a mesenchymal model of GSC (GBM10) with a high expression of Gal-3 but a very low expression of RAB10. Mechanistically, our results also identify β1 integrin as a critical partner of Gal-3/RAB10 as shown by macropinocytosis inhibition upon the genetic or pharmacologic knockdown of β1 integrin. The identification of this cluster in the mesenchymal GSCs sheds new light on macropinocytosis regulation in cancer cells.

With 70 members, RABs represent the largest family of the GTPase family (with 44 sub-families) that have been identified in humans. RABs widely differ among organisms and across phylogeny, reflecting the complexity of membrane transport events to which they contribute. However, only five RABs are consistently found in all eukaryotes, RAB1, RAB5, RAB6, RAB7, and RAB11, demonstrating their critical function.

In the context of our study, we have identified RAB10 as a critical molecule that is required for macropinocytosis-mediated survival. As for Gal-3, RAB10 knockdown induced a decrease in cell survival and macropinocytosis rate. However, unlike Gal-3 knockdown, our GSCs did not survive RAB10 knockdown and we had to generate 2D cell lines to be able to derive our cells, suggesting the critical function of RAB10 in GSC survival and, potentially, in GBM stemness. Several studies have shown that RAB10 was highly expressed in several cancers (such as liver and hepatocarcinoma, HCC)^46^. In HCC, a loss-of-function approach revealed several RAB10 downstream signaling pathways (such as InsR, Met/HGFR, c-Kit/SCFR, EphA3, EphB4, VEGFR2/KDR, Akt/PKB/Rac) that could be responsible for the regulation of cell survival. RAB10 can also regulate intracellular vesicle trafficking and has been shown to contribute to insulin-mediated translocation of glucose transporter type 4 (GLUT4) in adipocytes^47^. Moreover, RAB10 participates in the basement membrane protein trafficking to the lateral plasma membranes, promoting fibril formation^48^. Consistent with this idea, the authors showed that RAB10 over-expression increases both the amount of Collagen IV in the pericellular space and the fibrillar nature of the basement membrane. Of great interest, we found that our gene ontology enrichment analysis based on Gal-3^high^ *vs.* Gal-3^low^ in GBM patients highlighted genes involved in collagen catabolism/metabolism. Here, it is interesting to point out that, besides their role in macropinocytosis activity in GBM cells, Gal-3 and RAB10 might cooperate to regulate collagen metabolism in GBM.

The analysis of the Gal-3 interactome (via BioGRID) revealed more than 200 proteins that have been shown to bind to Gal-3. Among them, several proteins have been found in the reverse phase protein array from TCGA such as CAV1, HSP70, FN, PCNA, ITGB1, and ANXA1. For some other proteins, we did not find them in our IP-MS analysis but rather other members of their family like myosins (MYH9, MYH10), serpins (serpinA3, serpinB3, and serpin E2), and cell adhesion molecules (CAMs) such as MCAM and ICAM. Myosins are implicated in different forms of mobility including phagocytosis, vesicle trafficking or cell motility^49^. Gal-3 interaction with the unconventional MYH10 could be also involved in GBM cell motility, as MYH10 has been localized at the tips of filopodia and undergoes forward and retrograde movement within filopodia^50^. Moreover, MYH10 can bind to several RABs, including RAB10, and to transmembrane proteins such as CD44. Gal-3 is also known to bind to several CAMs such as laminin, Lamp, and Mac-2 binding proteins^24^. CAMs represent membrane receptors that mediate cell–cell and cell–matrix interactions. Many families including cadherins, selectins, integrins, or CD44 belong to CAMs and their expression is critical for transducing intracellular signals responsible for many cellular processes (such as migration, invasion, and angiogenesis). MCAM, also known as MUC18 or CD146, is upregulated in tumors of neuroectodermal origin and is involved in melanoma cell metastasis^51,52^. In melanoma, metastasis is promoted by Gal-3 interaction with MCAM, leading to cytokine secretion from vascular endothelial cells^53^. As for myosins, serpins, and integrins, we also showed that Gal-3 could interact with ICAM and desmoglein in GBM cells. Altogether, our study highlighted the complexity of the Gal-3 network in GBM.

In this era of precision medicine, it is clearly critical to being able to identify GBM patient populations that could be sensitive to targeted therapy^6–8^. By using a gene subset distinguishing between Gal-3^high^ *vs.* Gal-3^low^ co-expressed genes, we were able to split our small panel of GSCs between macropinocytosis-addicted *vs.* non-addicted. Moreover, by validating our transcriptomic signature on a different cohort, from the Mayo Clinic, we believe this signature could be expanded for clinical testing with clinically active Gal-3 inhibitors. Interestingly, some FDA approved Gal-3 inhibitors such as TD139 (from Galecto Biotech) in idiopathic pulmonary fibrosis or GM-CT-01 and GR-MD-02 (both from Galectin Therapeutics) in metastatic melanoma were found safe and well-tolerated by patients (clinical trials registered in clinicaltrials.gov: NCT03832946, NCT02117362, and NCT01723813, respectively). Therefore, based on our analysis of the TCGA dataset, we estimate that 20 % of GBM patients may show significant responses to agents targeting Gal-3/macropinocytosis. Remarkably, Gal-3 blockade by MCP appeared to be beneficial even for non-mesenchymal GSCs that are macropinocytosis non-addicted. Collectively, our study paves the way for considering macropinocytosis targeting in GBM as a promising strategy for clinical testing.

## Materials and methods

### GBM cell lines and patient-derived models

All cells were cultured in a standard tissue culture incubator maintained at 37 °C with 95% humidity and 5% CO_2_. Isolation of glioblastoma-initiating cells was performed as reported^54^. Ge269, 518, 835, 885, 898, 904, and 970.2 were cultured in Dulbecco’s modified Eagle’s medium (DMEM)/F12 with Glutamax supplemented with B27 supplement and b-FGF, EGF both at 10 ng/ml with antibiotics (GSC medium). GBM6, 10, 12, 39, 64, 116, 150, and 59 were requested from the Mayo Clinic Brain Tumor Patient-Derived Xenograft National Resource from Dr. Jann Sarkaria and cultured in GSC medium. We derived GDCs^36^. GDCs were maintained in DMEM-high glucose/glutamax, 10% fetal bovine serum, 1% penicillin/streptomycin. GSCs and GDCs were confirmed to be mycoplasma negative before experiments. GSCs gene expression has been assessed by quantitative real-time polymerase chain reaction^36^. All primers are listed in Supplementary Table 7.

### Orthotopic brain tumor xenograft model

All work was performed in accordance with the animal research committee of Geneva under the approved protocol (GE/38/20). 6-10-week-old female nu/nu immunocompromised mice weighing approximately 20–25 g were purchased from Charles River Labs, housed five per cage, and standard husbandry for specific pathogen-free provided by animal facility staff. Mice were allowed to acclimate for at least two weeks before any manipulation. Ge269 bearing genetic manipulations (shCtrl *vs.* shGal-3) were orthotopically transplanted following washing and resuspension in phosphate-buffered saline (PBS). Briefly, injection of tumor cells was made with a Hamilton syringe mounted on the frame, descending through the preformed hole to a depth of 3.6 mm into the putamen, a site far from the ventricles, and with little critical activity in the mouse. Injection of cells was at one microliter/minute. The syringe was withdrawn slowly over 5 min, the hole plugged with bone wax, and the scalp closed with two sutures and skin glue. Bodyweight was measured for each mouse pre-implantation, and then twice weekly for the duration of the experiments. Any mouse losing more than 15% of the pre-procedure body weight was euthanized immediately according to our protocol. Animals were monitored daily to evaluate tumor progression, and those exhibiting signs of morbidity and/or development of neurological symptoms were euthanized immediately (i.e., death was not used as an endpoint). Due to an absence of tumor in the brain after death, one animal was excluded from the shGal-3 group.

### Subcutaneous injection

Ge269, Ge518, and GBM10 GSCs (5 × 10^6^ tumor cells in 200 µl of PBS) were injected subcutaneously into the right flank of 6–10-week-old female nu/nu immunocompromised mice. Mice were treated with oral MCP (1% solution in the drinking water and replaced every 2 days). Tumor sizes were monitored three times per week with caliper until they were harvested (at 1000 mm^3^). Animals with a tumor size between 100 and 150 mm^3^ were then randomly allocated to each group, *n* = 3 for the vehicle (water) and *n* = 4 for the MCP group.

### Chemical inhibitors

MCP (PectaSol, Econugenics) was used at the concentration of 0.5% for cell viability and macropinocytosis assay for 3 days and 1 h, respectively. EIPA (5-(N-Ethyl-N-isopropyl)amiloride) and chlorpromazine were purchased from Sigma-Aldrich and used at the concentration of 0.5 µg ml^−1^ for cell viability and macropinocytosis assay for three days and 1 h, respectively.

### Genetic knockdown and expression constructs

Cells were infected with shRNAs for vector control (shCtrl, Open Biosystems), Gal-3 (Open Biosystems), RAB10 (Open Biosystems), PLX307 RAB10 (Addgene). Lentiviruses were produced^36^, by co-transfection of 293T cells with lentiviral backbone constructs and packaging vectors (ps-PAX2 and VSVG) using Lipofectamine 3000 (Thermo Fisher). The supernatant was collected 48 and 72 h post-transfection. Two weeks after puromycin selection, knockdown levels were confirmed by immunoblot analysis, and/or quantitative PCR and GSC were maintained in culture for one to two months at maximum as we observed a significant change in their growth otherwise. Gal-3 was ectopically expressed in GBM cells by transfecting the pEGFP-Gal-3 plasmid (Addgene).

### Macropinosome visualization and quantification

Macropinosome visualization experiments were performed^14^ for 2D culture and with some modifications for the 3D culture conditions. Briefly, cells were collected in 15 ml tubes in GSC medium. Macropinosomes were marked using a high-molecular TMR–dextran (Life Technologies) at a final concentration of 1 mg ml^−1^ for 1 h at 37 °C with or without EIPA/MCP pretreatment for 1 h at 0.5 µg ml^−1^ or 0.5%, respectively. At the end of the incubation period, cells were rinsed three times in cold PBS and immediately fixed in 2% cold formaldehyde. Cells were DAPI-treated for 10–15 min to stain nuclei and GSCs were coverslips mounted onto slides using Fluorsave (Calbiochem). Images were captured using an LSM800 Airyscan confocal microscope with 1.4 NA 63× oil-immersion lens using minimum pinhole (30 µm), or with Zeiss AxioCam microscope with 60×, 40×, and 20× objectives, leading to the calibration of 0.33 µm/pixel and 0.67 µm/pixel. Data were analyzed using the “Analyze Particles” feature in ImageJ (National Institutes of Health).

### Cell viability assay

GSCs were seeded at 20,000 cells per well in white 96-well plates in GSC medium. Cell viability was determined using CellTiter-Glo assay kit (Promega). Each condition consisted of, at least, three replicate wells and then luminescence read using a Cytation3 reader (BioTek). For cell viability assay under hypoxic conditions, GSCs were exposed either to atmospheric O_2_ conditions in a conventional hood and incubator (21%), or to 1% O_2_ by using the Ruskinn 300 InVivO2 hypoxia workstation (Baker) for 72 h. GSC medium was pre-equilibrated to 1% O_2_ by flushing with the corresponding gas mix.

### GSC 3D invasion in matrigel

GSC was seeded into 8-well chamber slides loaded with 150 µl of matrigel, on ice, after 1 h of EIPA or MCP pre-treatment. Totally, 150 µl of GSC medium was added on top of matrigel and GSC cell were incubated at 37 °C for 24 h to allow invasion assessment. Images were captured using EVOS microscope (life technologies) and analyzed using the “Analyze Particles” feature in ImageJ.

### Immunoblotting

Proteins were extracted in RIPA buffer and quantified using the Pierce BCA kit (Thermo Fisher). Totally, 20–30 µg of protein was boiled in NuPage buffer (Thermo Fisher) and loaded onto a denaturing SDS-polyacrylamide gel (10%), transferred to PVDF membranes and blotted with anti-mouse or -rabbit HRP-conjugated secondary antibodies (Bio-Rad). The following antibodies were used for immunoblotting: Gal-3 (Cell Signaling), RAB10 (Cell Signaling), β1 integrin (P4C10, Millipore), pAKT (Cell Signaling), AKT (Cell Signaling), pERK (Cell Signaling), ERK (Cell Signaling), and β-actin HRP (Sigma-Aldrich) as loading control. For protein expression analysis, expression was normalized to β-actin then compare to their respective control. For analysis of AKT and ERK activation, p-Akt and p-ERK were normalized to total AKT and total ERK, respectively, then compared to their control.

### Cell fractionation

Subcellular fractionation (SF) was performed following the protocol provided by Abcam (http://www.abcam.com/ps/pdf/protocols/subcellular_fractionation.pdf). Cells were lysed in sucrose–HEPES SF-based buffer for 30 min and cell lysates were then centrifuged. The pellet was resuspended in the nuclear buffer and the supernatant was ultra-centrifuged at 100,000*g* at 4 °C then collected as the cytosolic and membrane fraction. Finally, the supernatant was ultra-centrifuged at 100,000*g* at 4 °C, and the cytosolic fraction being in the supernatant. The pellet was resuspended with the SF buffer.

### Histological analysis, immunohistochemistry, and immunofluorescence

For immunohistochemical staining of formalin-fixed paraffin-embedded tissues, antigen retrieval was performed in citrate buffer at pH 6.0 and microwave for 15 min. Sections were blocked, then incubated overnight at 4 °C with primary antibody Gal-3 (Cell signaling), pAKT (Cell Signaling), AKT (Cell Signaling), Ki67 (Chemicon), and CD31 (Abcam) followed by biotin-conjugated anti-rabbit IgG and an avidin–biotin peroxidase detection system with 3,3′-diaminobenzidine substrate (Vector), then counterstained with hematoxylin (Sigma). Immunofluorescence of formalin-fixed paraffin-embedded GSCs was performed as reported^55^. The following primary antibodies against human antigens were used: rabbit anti-Gal-3 (Cell signaling) and mouse anti-RAB10 (Santa Cruz). The following fluorochrome-labeled secondary antibodies were used: Alexa Fluor (555 or 488)-labeled goat or donkey anti-mouse, or anti-rabbit antibodies. A Nikon Eclipse C1 Confocal microscope, as well as a Nikon Eclipse TE2000-E, were used for imaging.

### RAB10/Gal-3 cell-free binding assay

The RAB10/Gal-3 cell-free binding assay was performed as reported^14^. Briefly, 96-well plates were coated with purified human Gal-3 (Biolegend, 0.5 µg in 100 µl), incubated at 4 °C overnight, and then blocked with 50 mg/mL bovine serum albumin (BSA) for 90 min at 30 °C. After washing, recombinant human RAB10 (Prospec PRO-1361, from 0.25 to 0.5 µg/well) and MCP were combined and added for a total volume of 100 µl, then incubated for 4 h at 30 °C. Wells were washed, fixed with 2% PFA in PBS for 15 min at room temperature, washed, and then incubated with rabbit monoclonal RAB10 antibody (Abcam, 0.5 mg/mL diluted 1:100) for 1 h at RT. Wells were washed again and incubated with the secondary antibody (Life Technologies A11034, AF488 goat anti-rabbit IgG or A21206, AF488 donkey anti-rabbit IgG, both diluted 1:200) for 1 h at RT. Wells were washed three times and fluorescence read using a Cytation3 reader (BioTek) (ex.: 485 nm, em.: 538 nm) to quantify the binding of RAB10 to Gal-3.

### RAB10 kinetic assay

The RAB10 kinetic assay was performed using fluorescent BODIPY-GTP (Molecular Probes)^56,57^. Briefly, RAB10 proteins were diluted at 5 µM in assay buffer (1× TBS, 5 mM MgCl2, 0.1% BSA) and mixed in 384-well plates with indicated concentrations of GST-Gal-3 or GST as a control. Following 10 min incubation, the reaction was started by the addition of BODIPY-GTP to the final concentration of 0.5 µM, and recorded using a time-lapse fluorescence measurement in a Tecan Mplex plate reader (ex.:480 nm, em.:510 nm).

### Immunoprecipitation and immunoblots

Lysates from Ge518 and Ge269 shGal-3 and shCtrl were generated using an IP-MS kit (Life technologies). Immunoprecipitation of Gal-3 and RAB10 experiments were carried out according to the manufacturer’s instructions. For immunoblot analysis, 30 µg of protein was boiled in NuPAGE buffer and resolved on a pre-cast gel (Life technologies). Beads were resuspended in 100 μl of 6 M urea in 50 mM ammonium bicarbonate (AB). Totally, 2 μl of Dithioerythritol (DTE) 50 mM in distilled water were added and the reduction was carried out at 37 °C for 1 h. Alkylation was performed by adding 2 μl of iodoacetamide (400 mM in distilled water) for 1 h at room temperature in the dark. Urea concentration was reduced to 1 M by addition of 500 μl of AB and overnight digestion was performed at 37 °C with 5 μL of freshly prepared trypsin (Promega) at 0.1 μg/μl in AB. Supernatants were collected and completely dried under speed-vacuum. Samples were then desalted with a C18 microspin column (Harvard Apparatus, Holliston, MA, USA) according to manufacturer’s instructions, completely dried under speed-vacuum, and stored at −20 °C. LC–ESI–MS/MS was performed on a Q-Exactive Plus Hybrid Quadrupole-Orbitrap Mass Spectrometer (Thermo Fisher Scientific) equipped with an Easy nLC 1000 liquid chromatography system (Thermo Fisher Scientific).

### Analysis of IP–MS Data

Peak lists (MGF file format) were generated from raw data using the MS Convert conversion tool from ProteoWizard. The peaklist files were searched against the Human Reference Proteome database (Uniprot, 2018-06, 21044 entries) combined with an in-house database of common contaminants using Mascot (Matrix Science, London, UK; version 2.5.1). Trypsin was selected as the enzyme, with one potential missed cleavage. Precursor ion tolerance was set to 10 ppm and fragment ion tolerance to 0.02 Da. Carbamidomethyl of cysteine was specified as fixed modification. Deamidated of asparagine and glutamine, as well as oxidation of methionine, were specified as variable modifications. The Mascot search was validated using Scaffold 4.8.4 (Proteome Software). Peptide identifications were accepted if they could be established at greater than 6.0% probability to achieve an FDR less than 0.1% by the Peptide Prophet algorithm with Scaffold delta-mass correction^58^. Protein identifications were accepted if they could be established at greater than 80.0% probability to achieve an FDR less than 1.0% and contained at least two identified peptides. Protein probabilities were assigned by the Protein Prophet algorithm^59^. Proteins that contained similar peptides and could not be differentiated based on MS/MS analysis alone were grouped to satisfy the principles of parsimony. Proteins were annotated with GO terms from NCBI. A simple quantitative analysis based on the Normalized Total Spectra method was performed between the control and Gal-3 knockdown groups. A multiple *t* test with a significant level at *p* < 0.05 was applied for the triplicates where protein count was detected.

### Reverse transcription quantitative PCR (RT-qPCR)

Isolation of total RNA was performed by using RNeasy kit from Qiagen according to the manufacturer’s instructions. RNA concentration was determined using a spectrometer. Totally, 500 ng of total RNA was used to synthesize cDNA using a TAKARA kit according to manufacturer’s protocol. Primer sequences is described in Supplementary Table 7. Real-time PCR was performed using SYBR Green reagent at the genomic platform core facilities (University of Geneva). Efficacy tests have been performed, and all primers have been validated prior utilization. The relative level of each sample was normalized to, at least, two housekeeping genes (EEF1A1, ALAS1, TBP, and/or Tuba2). RT-PCR reactions were carried out in, at least, technical and biological triplicates, and the average cycle threshold (CT) values were determined. For evaluating Gal-3 expression after low oxygen exposure, GSCs were exposed to atmospheric O_2_ conditions in a conventional hood and incubator (21%), or to 1% O_2_ by using the Ruskinn 300 InVivO_2_ hypoxia workstation (Baker) for 48 h. GSC medium was pre-equilibrated to 1% O_2_ by flushing with the corresponding gas mix.

### Analysis of RNASeq data

The SR100—libraries TruSeqHT stranded—Illumina HiSeq 4000 was used and the sequencing quality control was done with FastQC v.0.11.5. The quality distribution along the reads plot validated for all samples. The reads were mapped with STAR aligner v.2.5.3a to the UCSC human hg38 reference. The average mapping rate was 92.97%. The differential expression analysis was performed with the statistical analysis R/Bioconductor package edgeR v. 3.18.1^60^. Briefly, the counts were normalized according to the library size and filtered. The genes having a count above one count per million reads (cpm) in at least four samples were kept for the analysis. The raw gene number of the set is 26’485. The poorly or not expressed genes were filtered out. The filtered data set consists of 12,737genes. The differentially expressed genes tests were done with exact test using a negative binomial distribution. The differentially expressed genes p-values are corrected for multiple testing error with a 5% FDR (false discovery rate). The correction used is Benjamini–Hochberg (BH). Then, the Figure was generated through Morpheus (https://software.broadinstitute.org/morpheus).

### In silico data analysis

As reported^36^, SurvExpress was also used to retrieve *p* value for Kaplan–Meier analysis of all galectins from TCGA dataset^61^. Survival analysis was performed for the Freije dataset. For TCGA, Rembrandt, IvyGAP, and Gravendeel datasets, data were obtained using GlioVis data portal for visualization (http://gliovis.bioinfo.cnio.es/) with a Log2 fold change of 1.5 and *p* value 0.05 for differentiate gene expression analysis. GlioVis uses Turkey’s Honest Significant Difference to evaluate the *p* value of the pairwise comparisons. For the reverse-phase protein array data for the TCGA dataset (Agilent-4502A platform), a cutoff of 0.11 was used. For evaluating our transcriptomic signature, genes expressed in the TCGA GBM RNASeq dataset were ranked using Nearest neighbors analysis (Pearson correlation) from Morpheus (https://software.broadinstitute.org/morpheus/) to check the similarity with LGALS3. Unsupervised hierarchical clustering (one minus Pearson correlation metric, average linkage method) was used to group genes (rows) and patients (columns) in the dataset (https://software.broadinstitute.org/morpheus/). The gene enrichment analysis was done using DAVID Bioinformatics resources^62,63^. Reactome was used to generate the protein enrichment analysis of the IP–MS data^64^.

### Statistics and reproducibility

Sample size and statistics for each experiment are provided in the Results section and Fig. Legends. Data shown are representative of results obtained for multiple experiments as noted in Fig. Legends. All statistical analyses were performed using one-way ANOVA, and two-way ANOVA (except when Student’s *t* test is noticed), with *p* < 0.05 considered significant. We also performed an analysis of variance applying a bivariate analysis. For in vivo experiments, all statistical analyses were carried out using Prism software (GraphPad). Chi-squared tests or *t* tests were used to calculate statistical significance.

### Data and software availability

The Supplemental Data includes nine supplemental figures and seven supplemental tables. Further information and requests for resources and reagents should be directed to and will be fulfilled by the Lead Contact and corresponding author, upon reasonable request.

### Reporting summary

Further information on research design is available in the Nature Research Reporting Summary linked to this article.

## Supplementary information

Supplementary Information

Description of Additional Supplementary Files

Supplementary Data 1

Reporting Summary

## Data Availability

The RNASeq data that support the findings of this study are available in Gene Expression Omnibus with the accession codes GSE173784.
