# Supplementary material for: Macropinocytosis requires Gal-3 in a subset of patient-derived glioblastoma stem cells
Source: Commun Biol. 2021 Jun 10;4:718. doi: 10.1038/s42003-021-02258-z (PMC8192788; doi:10.1038/s42003-021-02258-z)
Supplement: Supplementary file 3 — Supplementary Data 1 [file 42003_2021_2258_MOESM3_ESM.pdf]

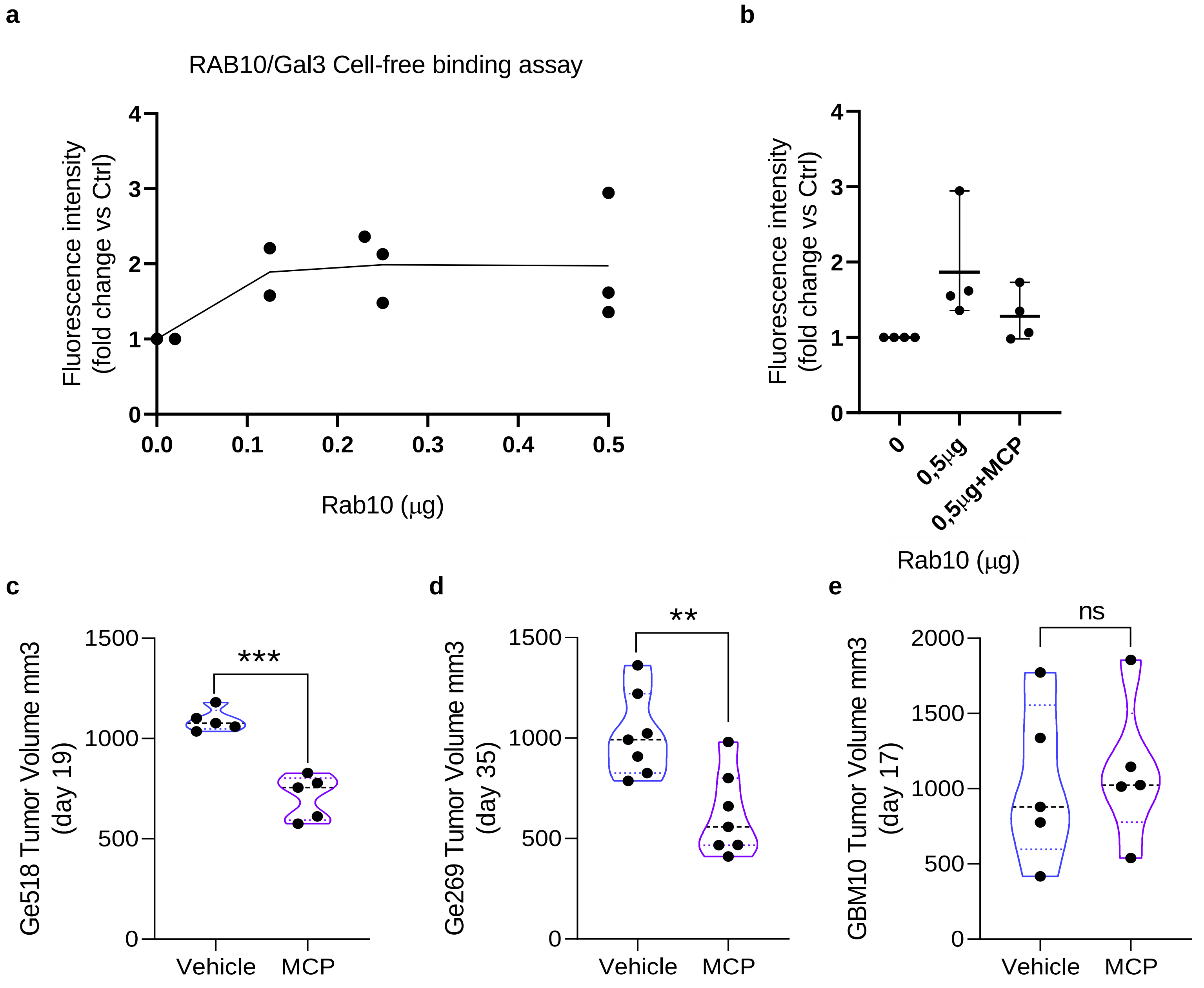

Supplementary data 1. The box-plot or dot-plot showing the experiment displayed in Figure 5e, supplementary figure 9c, and Figure7 d, e, and f.
