# Supplementary material for: Macropinocytosis requires Gal-3 in a subset of patient-derived glioblastoma stem cells
Source: Commun Biol. 2021 Jun 10;4:718. doi: 10.1038/s42003-021-02258-z (PMC8192788; doi:10.1038/s42003-021-02258-z)
Supplement: Supplementary file 4 — Reporting Summary [file 42003_2021_2258_MOESM4_ESM.pdf]

nature research

Corresponding author(s): Erika Cosset

Last updated by author(s): Mar 23, 2021

Reporting Summary

Nature Research wishes to improve the reproducibility of the work that we publish. This form provides structure for consistency and transparency in reporting. For further information on Nature Research policies, see our Editorial Policies and the Editorial Policy Checklist.

Statistics

For all statistical analyses, confirm that the following items are present in the figure legend, table legend, main text, or Methods section.

n/a	Confirmed	
☐	☒	The exact sample size (*n*) for each experimental group/condition, given as a discrete number and unit of measurement
☐	☒	A statement on whether measurements were taken from distinct samples or whether the same sample was measured repeatedly
☐	☒	The statistical test(s) used AND whether they are one- or two-sided<br>*Only common tests should be described solely by name; describe more complex techniques in the Methods section.*
☒	☐	A description of all covariates tested
☒	☐	A description of any assumptions or corrections, such as tests of normality and adjustment for multiple comparisons
☐	☒	A full description of the statistical parameters including central tendency (e.g. means) or other basic estimates (e.g. regression coefficient) AND variation (e.g. standard deviation) or associated estimates of uncertainty (e.g. confidence intervals)
☒	☐	For null hypothesis testing, the test statistic (e.g. *F*, *t*, *r*) with confidence intervals, effect sizes, degrees of freedom and *P* value noted<br>*Give P values as exact values whenever suitable.*
☒	☐	For Bayesian analysis, information on the choice of priors and Markov chain Monte Carlo settings
☐	☒	For hierarchical and complex designs, identification of the appropriate level for tests and full reporting of outcomes
☒	☐	Estimates of effect sizes (e.g. Cohen's *d*, Pearson's *r*), indicating how they were calculated

Our web collection on statistics for biologists contains articles on many of the points above.

Software and code

Policy information about availability of computer code

Data collection	For TCGA, Rembrandt, IvyGAP and Gravendeel dataset, data were obtained using GlioVis data portal for visualization (http://gliovis.bioinfo.cnio.es/) with a Log2 fold change of 1.5 and pValue 0.05 for differentiate gene expression analysis. GlioVis uses the Turkey's Honest Siginificant Difference to evaluate the p value of the pairwise comparisons. For the reverse phase protein array data for TCGA dataset (Agilent-4502A platform), a cutoff of 0.11 was used. For evaluating our genetic signature, genes expressed in the TCGA GBM RNASeq dataset were ranked using Nearest neighbors analysis (Pearson correlation) from morpheus (https://software.broadinstitute.org/morpheus/) to check the similarity with LGALS3. Unsupervised hierarchical clustering (one minus pearson correlation metric, average linkage method) was used to group genes (rows) and patients (columns) in the dataset (https://software.broadinstitute.org/morpheus/). The gene enrichment analysis was done using DAVID Bioinformatics resources.
Data analysis	Analysis of RNASeq data.<br>The SR100 – libraries TruSeqHT stranded – Illumina HiSeq 4000 was used and the sequencing quality control was done with FastQC v.0.11.5. The quality distribution along the reads plot validated for all samples. The reads were mapped with STAR aligner v.2.5.3a to the UCSC human hg38 reference. The average mapping rate was 92.97%. The differential expression analysis was performed with the statistical analysis R/Bioconductor package edgeR v. 3.18.1. Briefly, the counts were normalized according to the library size and filtered. The genes having a count above one count per million reads (cpm) in at least four samples were kept for the analysis. The raw gene number of the set is 26'485. The poorly or not expressed genes were filtered out. The filtered data set consists of 12'737genes. The differentially expressed genes tests were done with exact Test using a negative binomial distribution. The differentially expressed genes p-values are corrected for multiple testing error with a 5% FDR (false discovery rate). The correction used is Benjamini-Hochberg (BH). Then, Fig. was generated through Morpheus (https://software.broadinstitute.org/morpheus).

For manuscripts utilizing custom algorithms or software that are central to the research but not yet described in published literature, software must be made available to editors and reviewers. We strongly encourage code deposition in a community repository (e.g. GitHub). See the Nature Research guidelines for submitting code & software for further information.

Data

Policy information about availability of data

All manuscripts must include a data availability statement. This statement should provide the following information, where applicable:

- Accession codes, unique identifiers, or web links for publicly available datasets
- A list of figures that have associated raw data
- A description of any restrictions on data availability

Provide your data availability statement here.

Field-specific reporting

Please select the one below that is the best fit for your research. If you are not sure, read the appropriate sections before making your selection.

☒ Life sciences ☐ Behavioural & social sciences ☐ Ecological, evolutionary & environmental sciences

For a reference copy of the document with all sections, see nature.com/documents/nr-reporting-summary-flat.pdf

Life sciences study design

All studies must disclose on these points even when the disclosure is negative.

Sample size	For the mice experiment, our group size has been calculated based on statistical guidance on the www.3rs-reduction.co.uk/ website. For the in vitro experiments, all experiments were repeated at least three times.
Data exclusions	Due to an absence of tumor in the brain after death, one animal was excluded from the shGal-3 group.
Replication	For the in vitro experiments, all experiments were repeated at least three times.
Randomization	Animals with a tumor size between 100 and 150mm3 were then randomly allocated to the control or the treated group.
Blinding	The investigators were blinded to allocation during outcome assessment.

Reporting for specific materials, systems and methods

We require information from authors about some types of materials, experimental systems and methods used in many studies. Here, indicate whether each material, system or method listed is relevant to your study. If you are not sure if a list item applies to your research, read the appropriate section before selecting a response.

Materials & experimental systems

n/a	Involved in the study	
☐	☒	Antibodies
☐	☒	Eukaryotic cell lines
☒	☐	Palaeontology and archaeology
☐	☒	Animals and other organisms
☒	☐	Human research participants
☒	☐	Clinical data
☒	☐	Dual use research of concern

Methods

n/a	Involved in the study	
☒	☐	ChIP-seq
☒	☐	Flow cytometry
☒	☐	MRI-based neuroimaging

Antibodies

Antibodies used	Gal-3 (Cell signaling), pAKT (Cell Signaling), AKT (Cell Signaling), Ki67 (Chemicon), and CD31 (Abcam), RAB10 (Cell Signaling), β1 integrin (P4C10, Millipore), pAKT (Cell Signaling), AKT (Cell Signaling), pERK (Cell Signaling), ERK (Cell Signaling), and β-actin HRP (Sigma-Aldrich)
Validation	All antibodies have been validated for the readouts for which their have been used according to the manufacturer's recommendation.

Eukaryotic cell lines

Policy information about cell lines

Cell line source(s)	Patient-derived cells

Authentication	N/A
Mycoplasma contamination	GSCs and GDCs were confirmed to be mycoplasma negative before experiments.
Commonly misidentified lines (See ICLAC register)	N/A

Animals and other organisms

Policy information about studies involving animals; ARRIVE guidelines recommended for reporting animal research

Laboratory animals	6-10-week-old female nu/nu immunocompromised mice
Wild animals	N/A
Field-collected samples	Mice were purchased from Charles River Labs, housed five per cage, and standard husbandry for specific pathogen free (SPF) provided by animal facility staff. Mice were allowed to acclimate for at least two weeks before any manipulation.
Ethics oversight	All work was performed in accordance with the animal research committee of Geneva under the approved protocol (GE/38/120).

Note that full information on the approval of the study protocol must also be provided in the manuscript.
