# Supplementary material for: Macropinocytosis requires Gal-3 in a subset of patient-derived glioblastoma stem cells
Source: Commun Biol. 2021 Jun 10;4:718. doi: 10.1038/s42003-021-02258-z (PMC8192788; doi:10.1038/s42003-021-02258-z)
Supplement: Supplementary file 1 — Supplementary Information [file 42003_2021_2258_MOESM1_ESM.pdf]

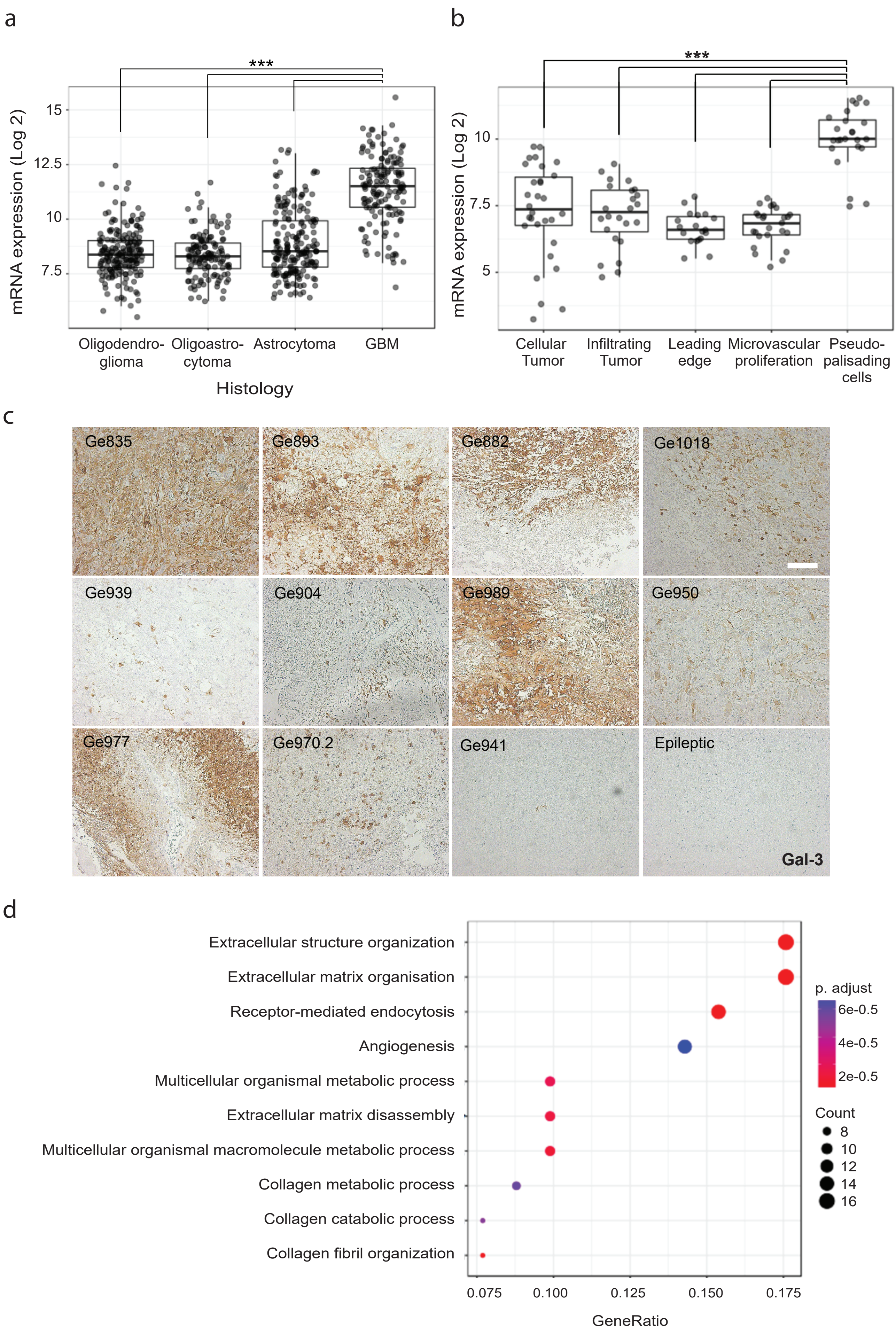

Supplementary Figure 1. Gal-3 levels correlate with poor survival and macropinocytosis rate in GBM. (a) Gal-3 expression increases with grade and is highly expressed in GBM. The Tukey's Honest Significant Difference was used to evaluate the p-value of the pairwise comparisons in TCGA_GBMLGG dataset. ***p<0.001. (b) Gal-3 expression is higher in pseudopalisading cells as shown by Ivy GAP dataset (extracted from Gliovis). The Tukey's Honest Significant Difference was used to evaluate the p-value of the pairwise comparisons. ***p<0.001. (c) Histological analysis of GBM tumor biopsies. Tumors were stained for Gal-3, and counterstained with hematoxylin. The biopsies Ge835, Ge893, Ge882 and Ge1018 are enriched for mesenchymal genes, Ge939, Ge904, Ge977 are enriched for classical genes, Ge941, Ge950 for proneural genes and Ge970.2 for neural genes. Scale bar, 50μm. (d) Gene ontology enrichment analysis of Gal-3 from Rembrandt dataset. Log2 fold change of 1.5 and p value of 0.05 were used as filter data.

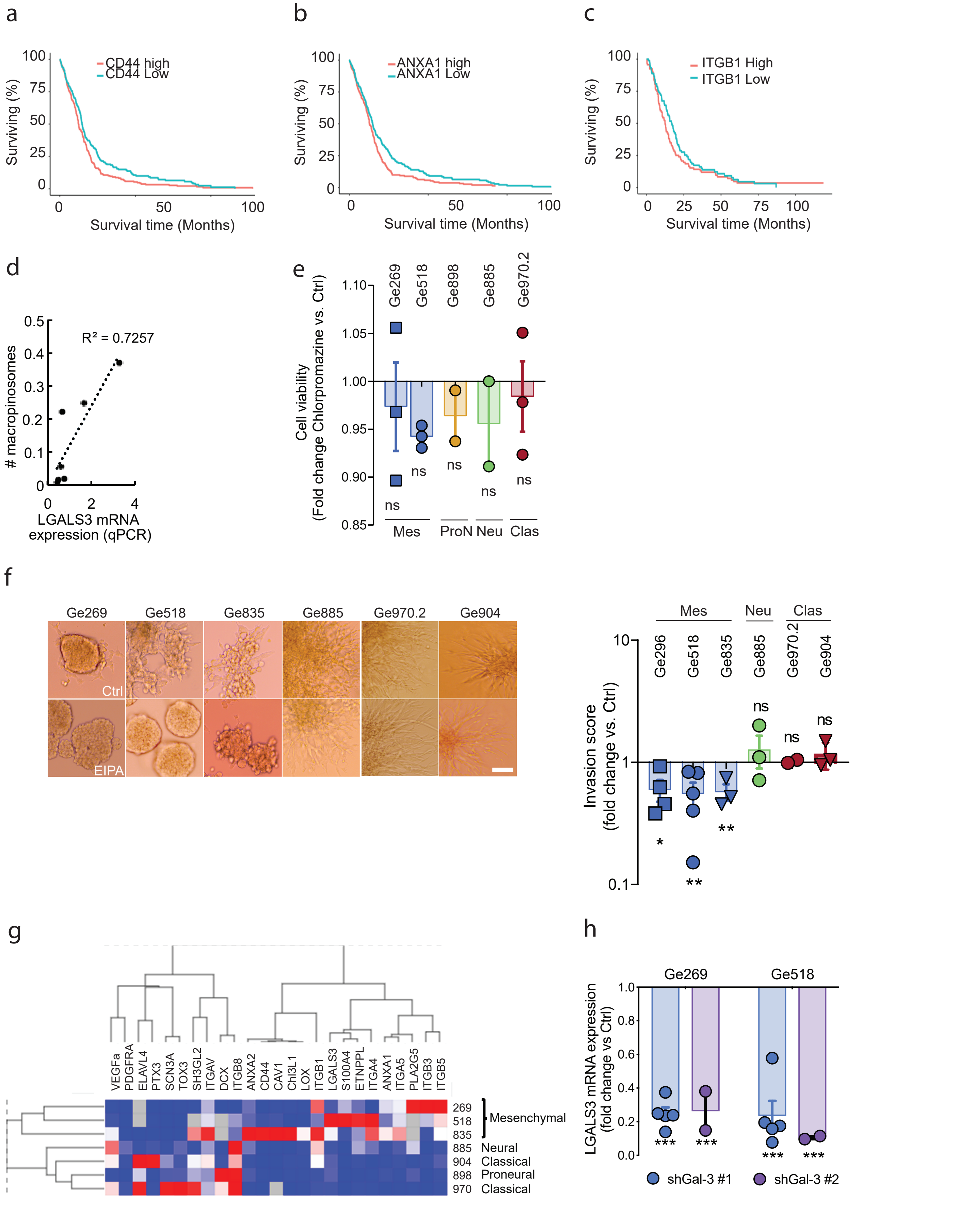

Supplementary Figure 2. Gal-3 expression is associated with the mesenchymal subtype. (a) Kaplan-Meier analysis of Rembrandt dataset for CD44 expression (n = 197 CD44low, n = 200 CD44high; Log-rank p= 0.0052). (b) Kaplan-Meier analysis of Rembrandt dataset for ANXA1 expression (n = 182 ANXA1low, n = 133 ANXA1high; Log-rank p value (p) 0.0032). (c) Kaplan-Meier analysis of Rembrandt dataset for ITGB1 expression (n = 92 ITGB1low, n = 89 ANXA1high; Log-rank p value (p) 0.17). (d) Correlation of the macropinocytosis activity to Gal-3 mRNA expression evaluated by TMR-dextran uptake and qPCR, respectively. (e) Effect of chlorpromazine on cell viability measured by CellTiter-Glo in GSCs (n=3-6). (f) Effect of EIPA on GSC cell invasion in 3D. The distance is measured as described in the method section (n=3-5). (g) The differential gene expression analysis based on Gal-3high vs Gal-3low extracted from the TCGA dataset (Log2 fold change = 2; p value = 0.05) was used to generate a list of Gal-3/survival-associated genes predicted to identify the addicted vs non-addicted phenotype. Heat map represents fold-changes of mRNA expression measured by qPCR for the identified genes (red, FC>1; blue, FC<0.8). (h) Gal-3 mRNA expression was determined by qPCR in Ge269 and Ge518 transduced by shCtrl or shGal-3 (n=2-5). Data are represented as mean ± SEM (*p<0.05, **p<0.01 and ***p<0.001), 2-way ANOVA, Sidak's adjusted p-value. ns = non-significant.

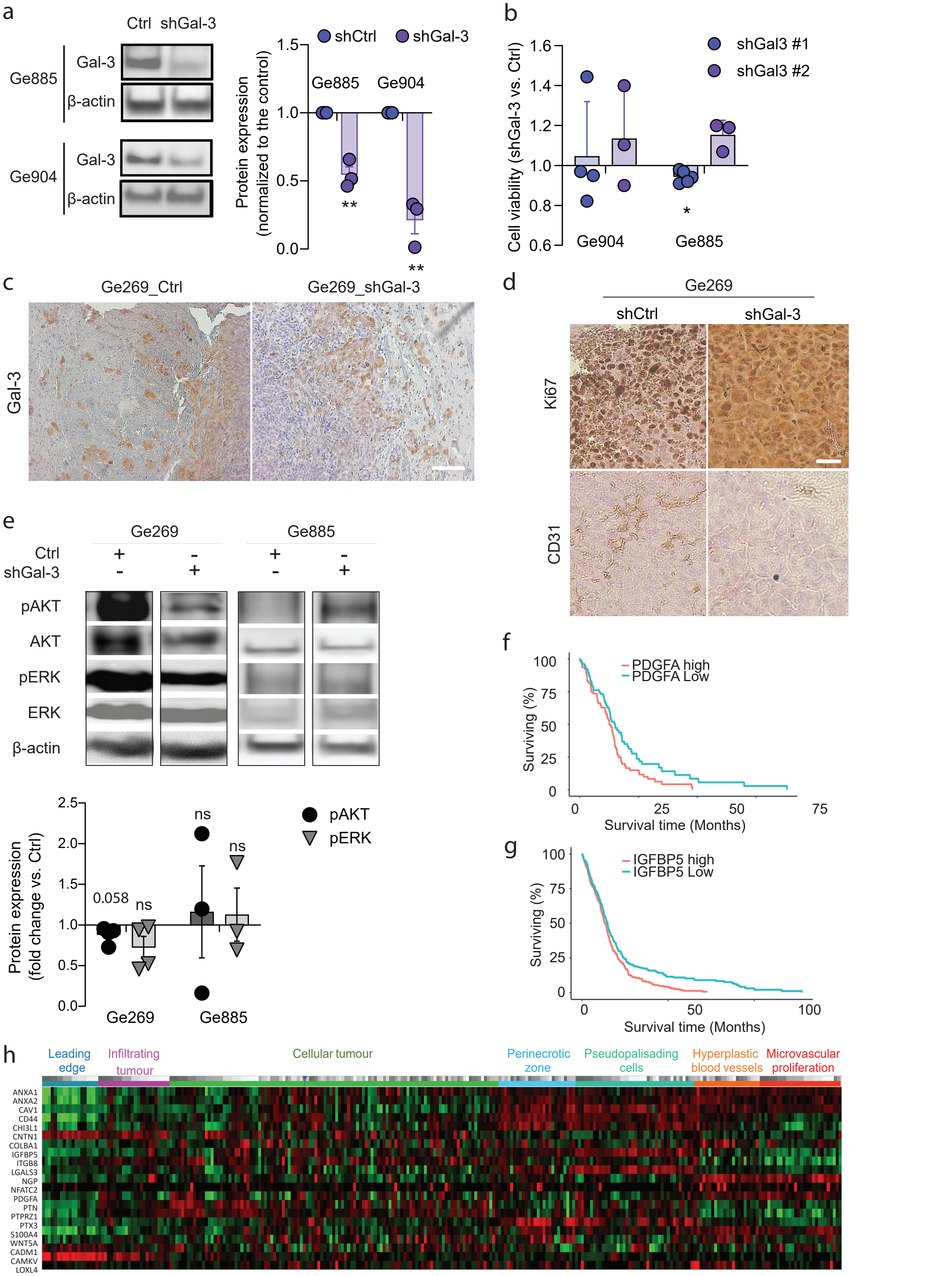

Supplementary Figure 3. Histological analysis of Ge269 in mice and correlation of PDGFA and IGFBP5 expression with poor survival and pseudopalisading area. (a) Western blot showing the expression of Gal-3 in shCtrl vs shGal-3, in Ge885 and Ge904 GSCs. Quantification of Gal-3 expression is shown on the right. (n=3). (b) Cell viability of Ge 904 and Ge885 in shCtrl vs shGal-3, measured by Celltiter-Glo. (n=3-5). (c) Histological analysis of Ge269 shCtrl and shGal-3 tumors. Tumors were stained for Gal-3, and counterstained with hematoxylin. (d) Histological analysis of Ge269 shCtrl and shGal-3. Tumors were stained for Ki-67, CD31, and counterstained with hematoxylin. (e) Western blot showing the expression of the indicated proteins in Ge269 and Ge885 shGal-3 vs shCtrl. Histogram represents the expression of pAKT and pERK normalized to AKT and ERK, respectively. Student t-test was applied. ns = non-significant. (f) Kaplan-Meier analysis of TCGA dataset for PDGFα expression (n = 264 PDGFαlow, n = 261 PDGFαhigh; Log-rank p= 0.0067. (g) Kaplan-Meier analysis of TCGA dataset for IGFBP5 expression (n = 263 IGFBP5low, n = 262 IGFBP5high; Log-rank p= 0.0031. (h) ANXA1, ANXA2, CAV1, CHI3L1, IGFBP5 and PTX3 expression are higher in peudopalisading cells as shown by Ivy GAP dataset. Data are represented as mean ± SEM (*p<0.05, **p<0.01 and ***p<0.001), 2-way ANOVA, Sidak's adjusted p-value. ns = non-significant.

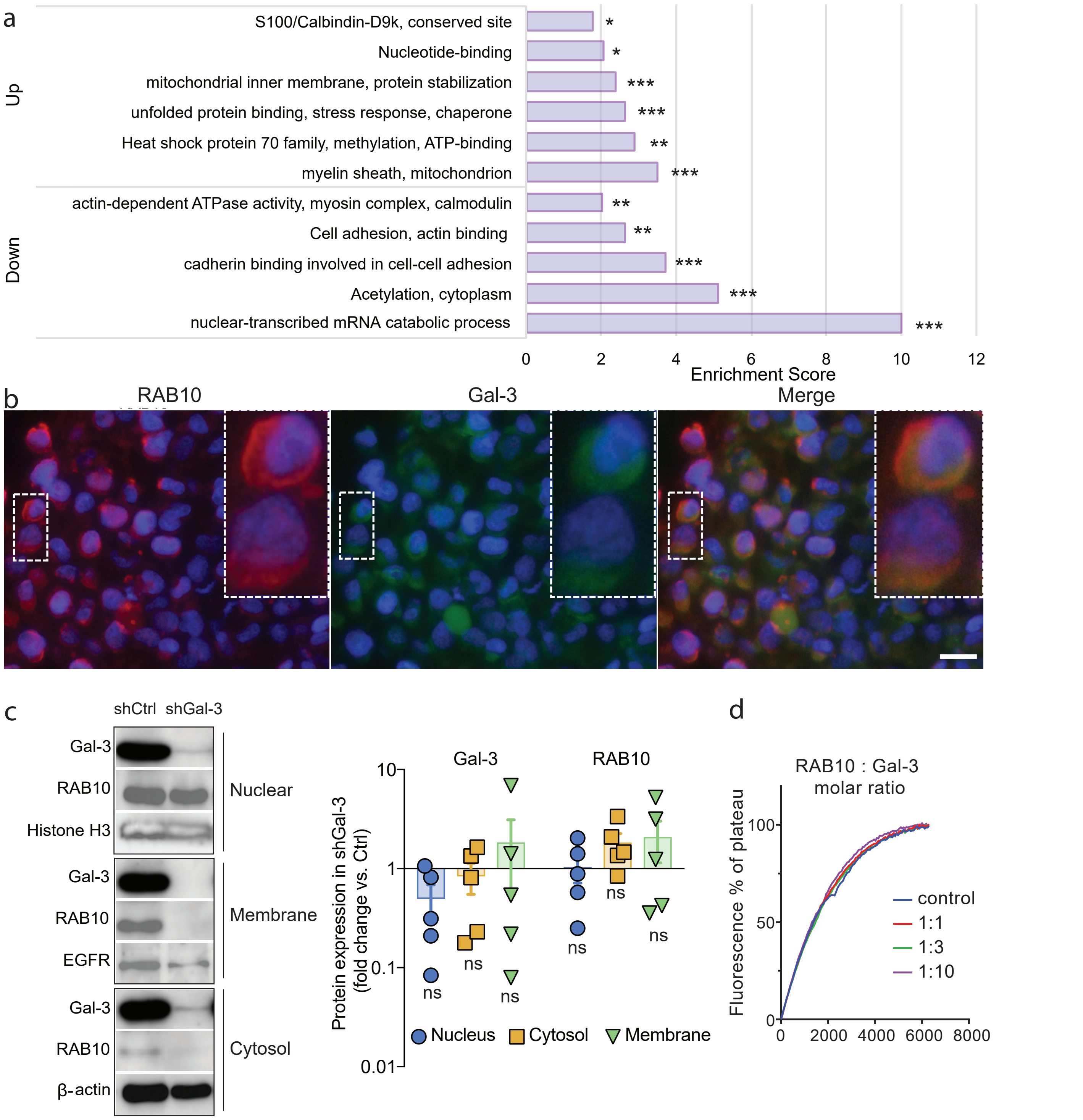

Supplementary Figure 4. Gal-3 and RAB10 co-localization at the plasma membrane. (a) Protein ontology enrichment analysis of shGal-3 compare to their control after IP-MS analysis. *p<0.05, **p<0.01 and ***p<0.001. (b) The colocalization of Gal-3 (stained in green) and RAB10 (in red) was visualized by immunofluorescence. Cell nuclei were stained with DAPI (4, 6-diamidino-2-phenylindole). Scale bar, 20µm. (c) Immunoblots showing subcellular fractionation of Ge518 shCtrl vs shGal-3. Histogram represents Gal-3 and RAB10 normalized to a loading control related to each fraction and determined by densitometry analysis (n=5). (d) Graph depicts a BODIPY-GTP uptake assay of GTP-loaded Gal-3 and purified RAB10 at the indicated molar ratio. (e) Western blot showing the expression of Gal3 and RAB10 in Ge904 vs Ge904 pEGFP-Gal-3/PLX307-RAB10 (Ge904 PLX) cells. Histogram represents the expression of Gal3 and RAB10 normalized to Actin. (f) TMR dextran uptake (red) by Ge904 vs Ge904 PLX. Dapi staining in blue. Histogram represents the number of macropinosomes in Ge904 PLX compared to the Ge904 Ctrl. Data are represented as mean ± SEM (*p<0.05, **p<0.01 and ***p<0.001), 2-way ANOVA, Sidak's adjusted p-value. ns = non-significant.

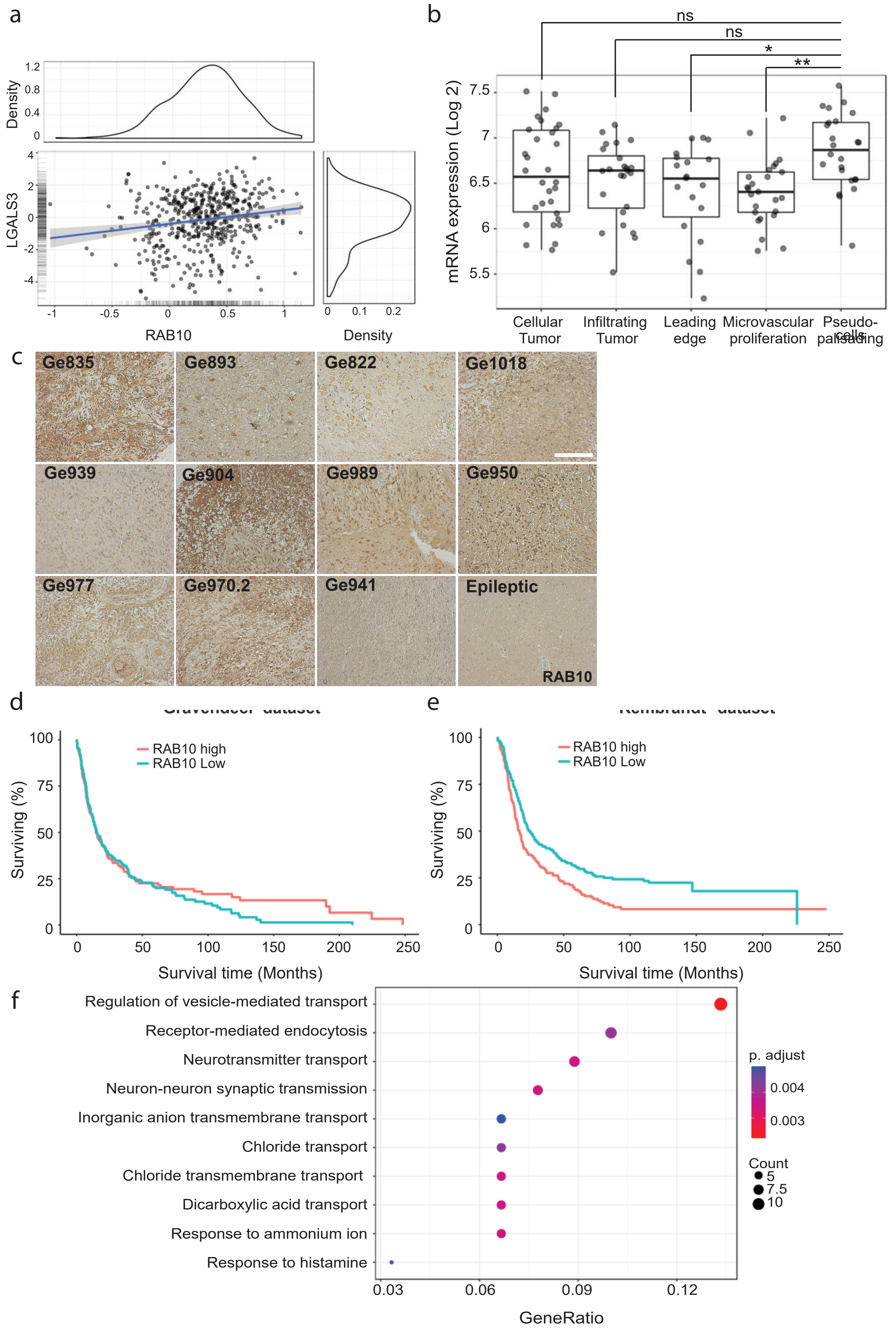

Supplementary Figure 5. RAB10 expression is correlated with Gal-3 but not consistently correlated with patient survival. (a) Gal-3 and RAB10 are correlated genes as evaluated by Pearson's correlation in TCGA dataset (for Agilent 4502A). p <0.0001. (b) RAB10 expression is higher in peudopalisading cells as shown by Ivy GAP dataset (extracted from Gliovis). Tukey's honest significant difference ***p<0.001; **p<0.01; *p<0.05; ns = not significant. (c) Histological analysis of GBM tumor biopsies. Tumors were stained for RAB10, and counterstained with hematoxylin. Scale bar, 50µm. (d) Kaplan-Meier analysis of TCGA dataset for RAB10 expression (n = 134 RAB10low, n = 137 RAB10high; Log-rank p= 0.37. (e) Kaplan-Meier analysis of Rembrandt dataset for RAB10 expression (n = 200 RAB10low, n = 197 RAB10high; Log-rank p= 0.0004. (f) Gene ontology enrichment analysis of RAB10.

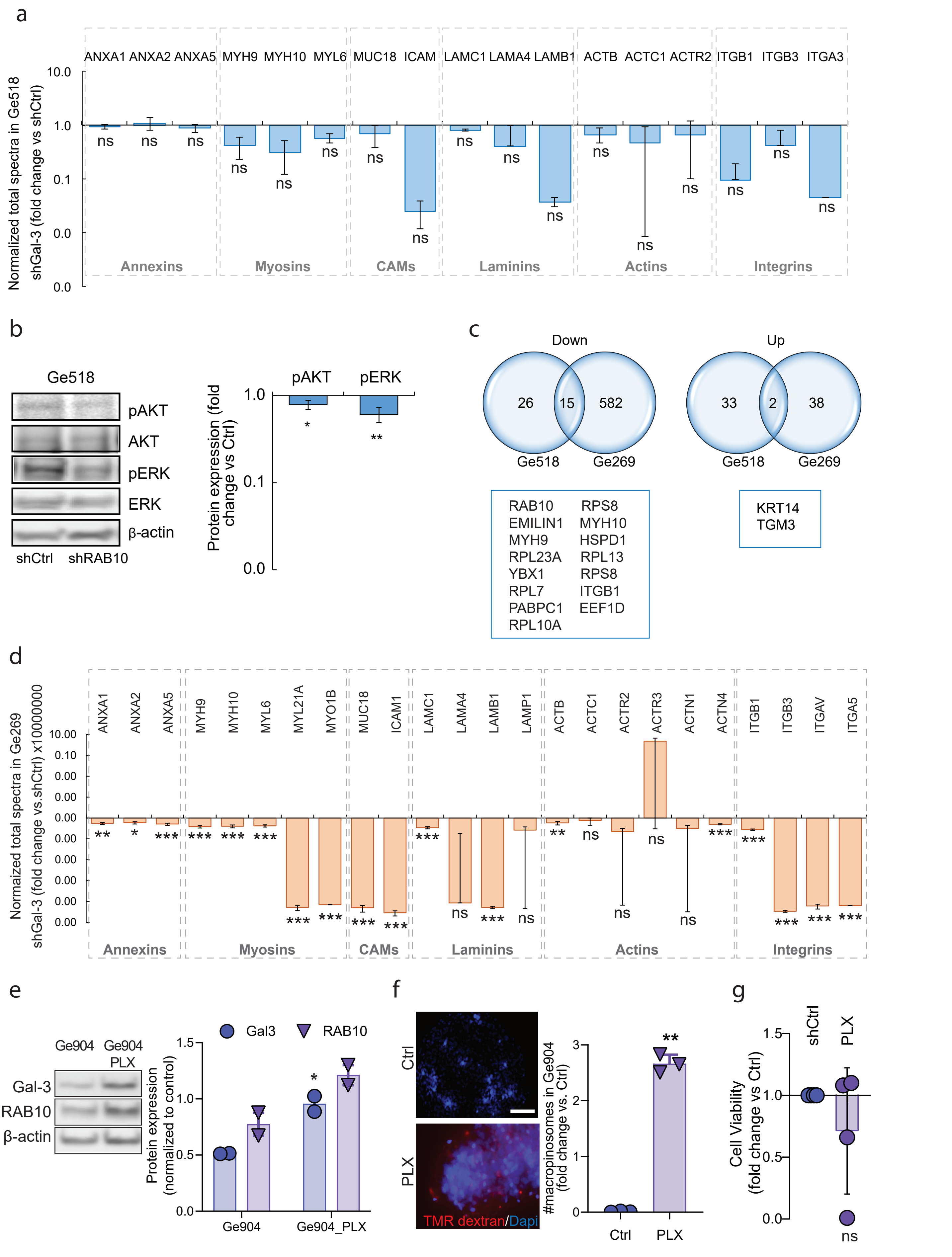

Supplementary Figure 6. RAB10 knockdown induces a decrease of AKT and ERK activation. (a) Histograms representing the fold change for normalized total spectra for annexins, myosins, CAMs, laminins, actins, and integrins identified by IP-MS analysis in Ge518 and Ge269 for shCtrl versus shGal-3. A multiple t-test was used when protein count was detected in the triplicates. ***p<0.001; **p<0.01; *p<0.05; ns= not significant. (b) Venn Diagram showing the number of proteins whose expression is downregulated or upregulated in Ge518 and Ge269, according to IP-MS. (c) Immunoblots showing expression of indicated proteins when RAB10 is knocked down in Ge518. Histograms represent the fold change of protein expression determined by densitometry analysis (n=3). (d) Histogram representing the tumor volume at day 28 post-injection of Ge269 cells in mice. ***p<0.001. (e) Histogram representing the tumor volume at day 19 post-injection of Ge518 cells in mice. **p<0.01. (f) Histogram representing the tumor volume at day 19 post-injection of GBM10 cells in mice. ns = not significant.

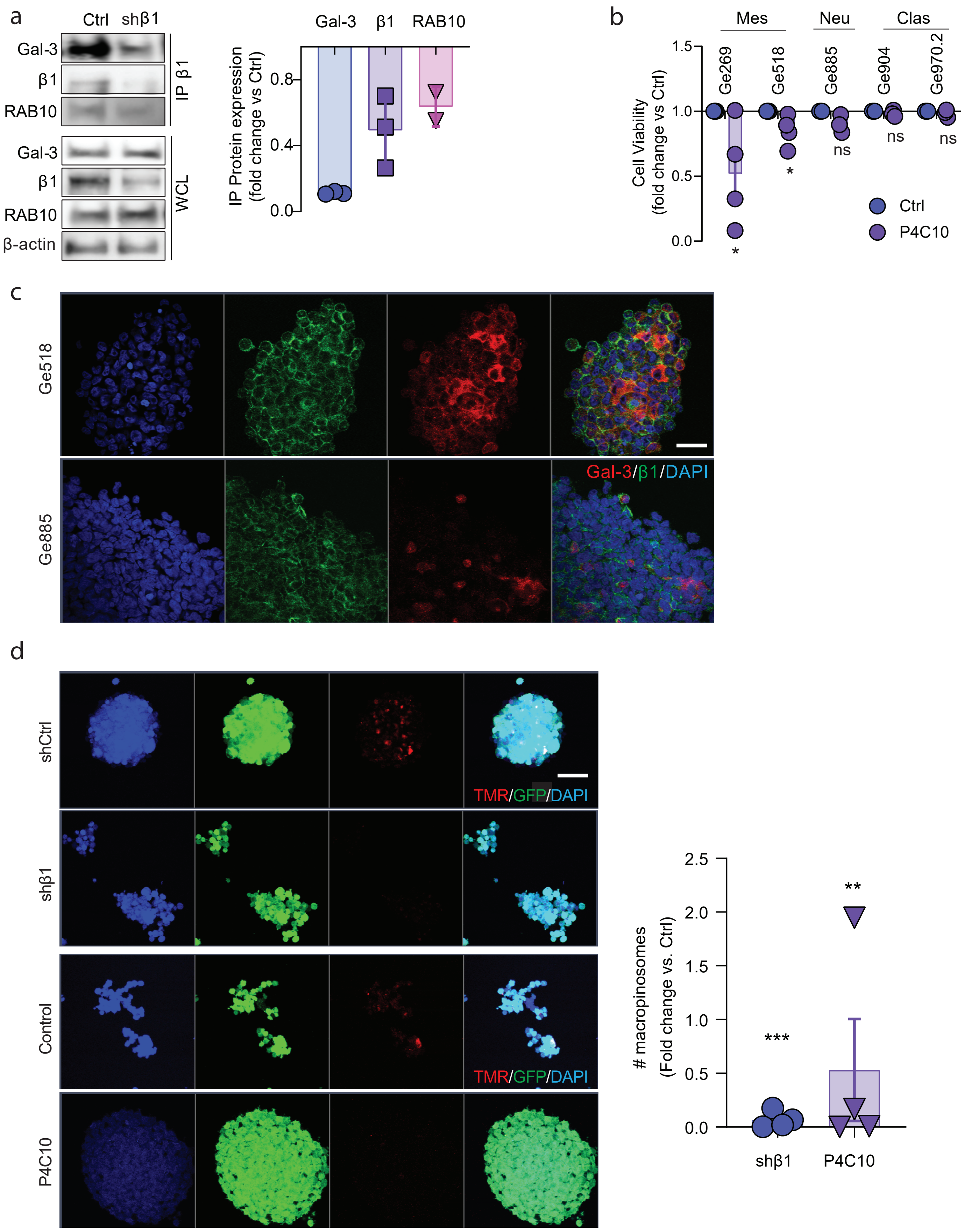

Supplementary Figure 7. The genetic or pharmacologic knock down of β1 integrin disrupt its interaction with Gal-3 and induces an inhibition of macropinocytosis-mediated cell survival. (a) Western blot showing the expression of Gal-3, β1 and RAB10 after immunoprecipitation of β1. Lower panel: Western blot showing the expression of Gal-3, β1, RAB10 and Actin in WCL (whole cell lysate). Histogram represents the expression of Gal3, β1 and RAB10 after IP in shβ1, normalized to the IP control condition. (b) Cell viability of Ge269, Ge518, Ge885, Ge904 and Ge970.2 cells treated with P4C10. The result is normalized to the control. (c) Immunodetection of Gal-3 (red), β1 (green), Dapi nuclear staining (blue) in Ge518 and Ge885 GSCs. (d) TMR dextran uptake (red) by Ge269 in shβ1 condition and after P4C10 treatment. GFP staining in green. Dapi staining in blue. Histogram represents the number of macropinosomes in Ge269 shβ1 and under P4C10 treatment compared to their respective controls. *$p<0.05$, **$p<0.01$ and ***$p<0.001$.

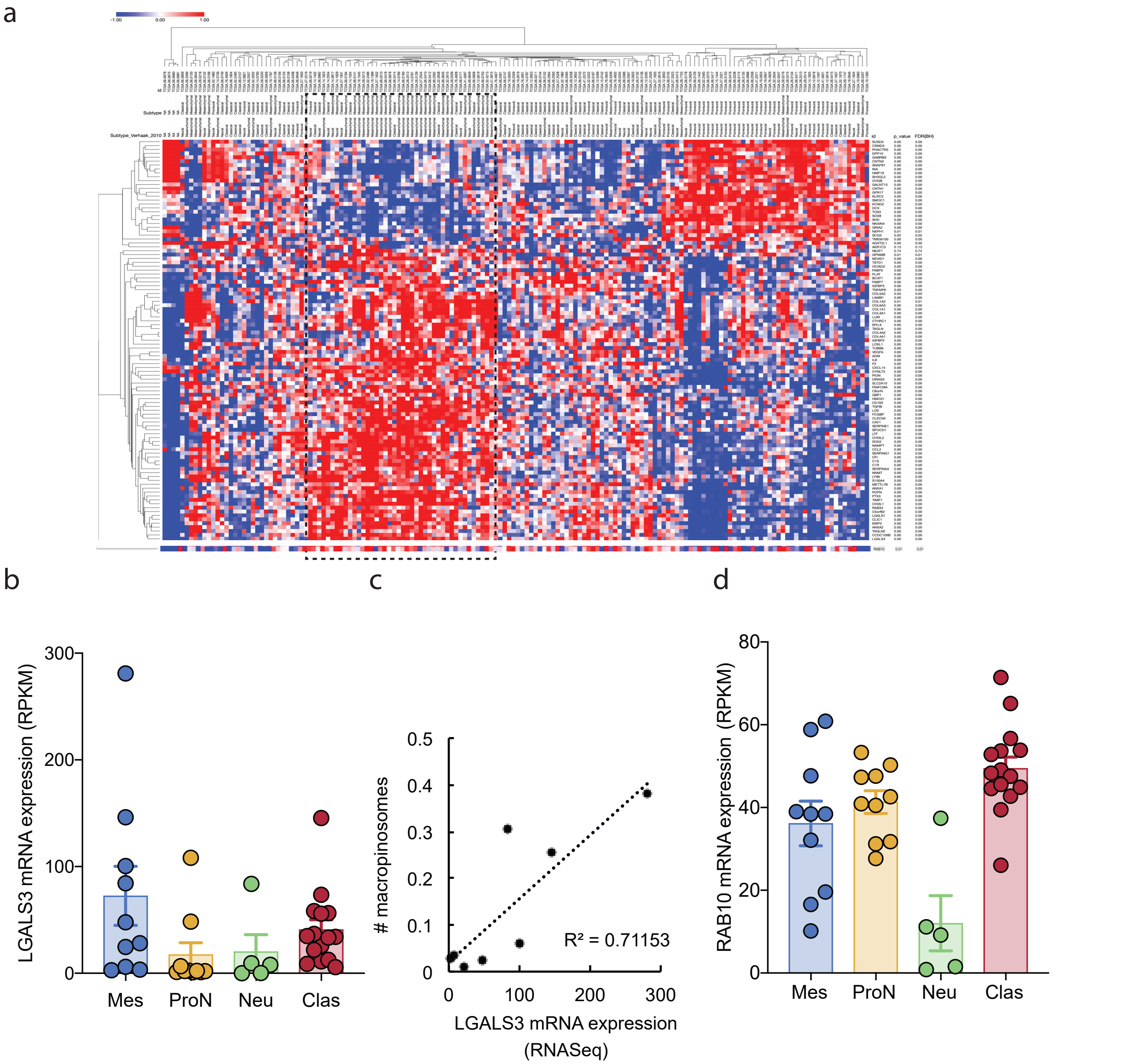

Supplementary Figure 8. Macropinocytosis blockade can be predicted by a transcriptomic signature. (a) Heatmap showing the transcriptomic signature applying on TCGA dataset. Black square dot represents Gal-3/macropinocytosis addicted patients. (b) Gal-3 mRNA expression was determined by RNASeq (normalized as RPKM) in Mayo Clinic GSCs. (c) Correlation of the macropinocytosis activity to Gal-3 mRNA expression evaluated by TMR-dextran uptake and RNASeq, respectively. (d) RAB10 mRNA expression was determined by RNASeq (normalized as RPKM) in Mayo Clinic GSCs.

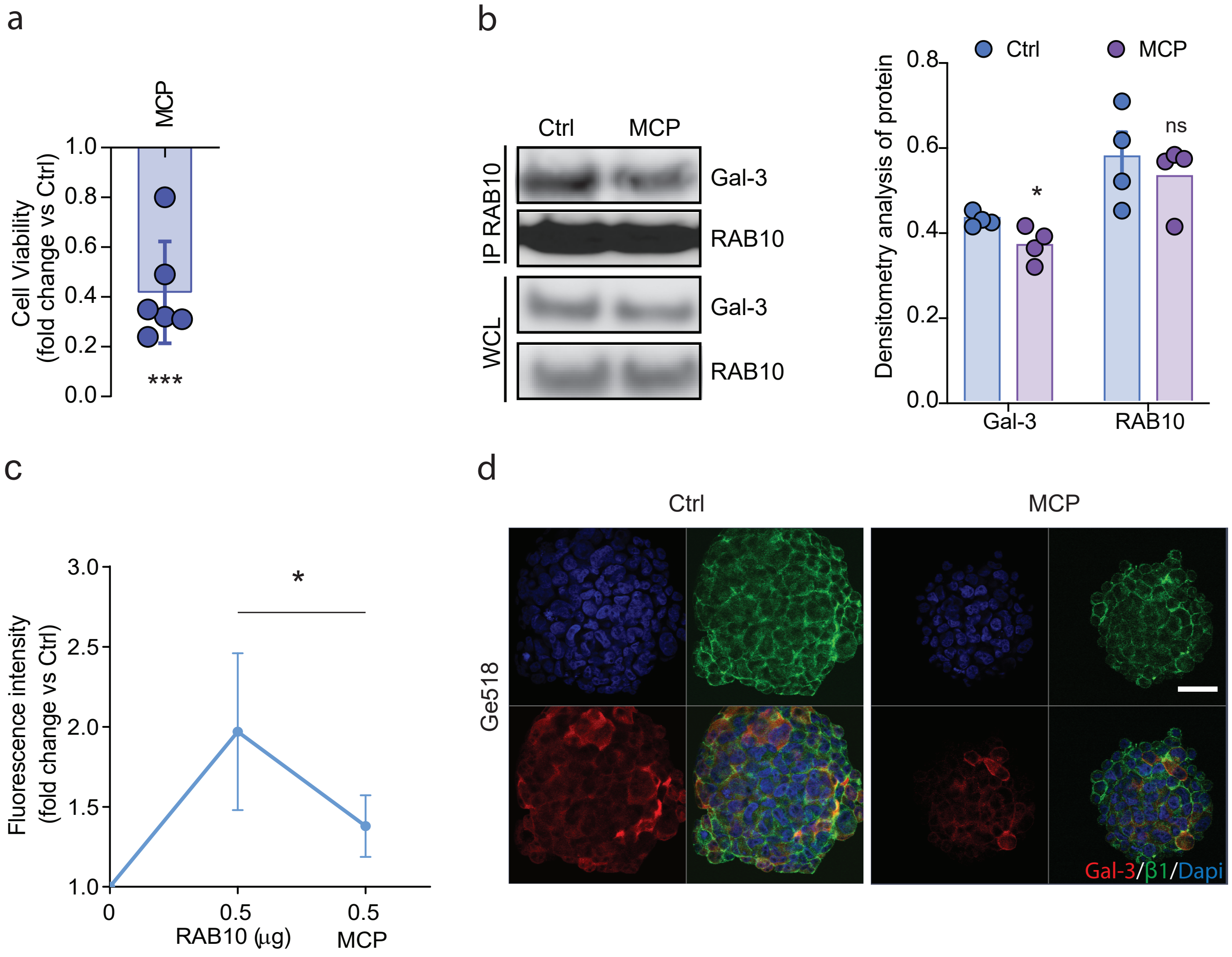

Supplementary Figure 9. MCP treatment induced a decrease of macropinocytosis-mediated GSC survival and disrupted Gal-3/RAB10 interaction. (a) Cell viability of Ge904 ectopically expressing Gal-3 and RAB10 treated with MCP. The result is normalized to the control. (n=4-6) *p<0.05, **p<0.01 and ***p<0.001. (b) Immunoblot analysis of RAB10 immunoprecipitation from Ge518 cells treated with MCP for 12 hours. Histograms represent the fold increase of Gal-3 and RAB10 expression determined by densitometry analysis (n=4). WCL, whole-cell lysate. (c) Effect of MCP on Gal-3/ RAB10 binding measured by a cell-free binding assay (n=3). (d) The colocalization of Gal-3 (stained in red) and β1 integrin (in green) was visualized by immunofluorescence. Cell nuclei were stained with Dapi (4, 6-diamidino-2-phenylindole). Scale bar, 20µm.

Figure 2A

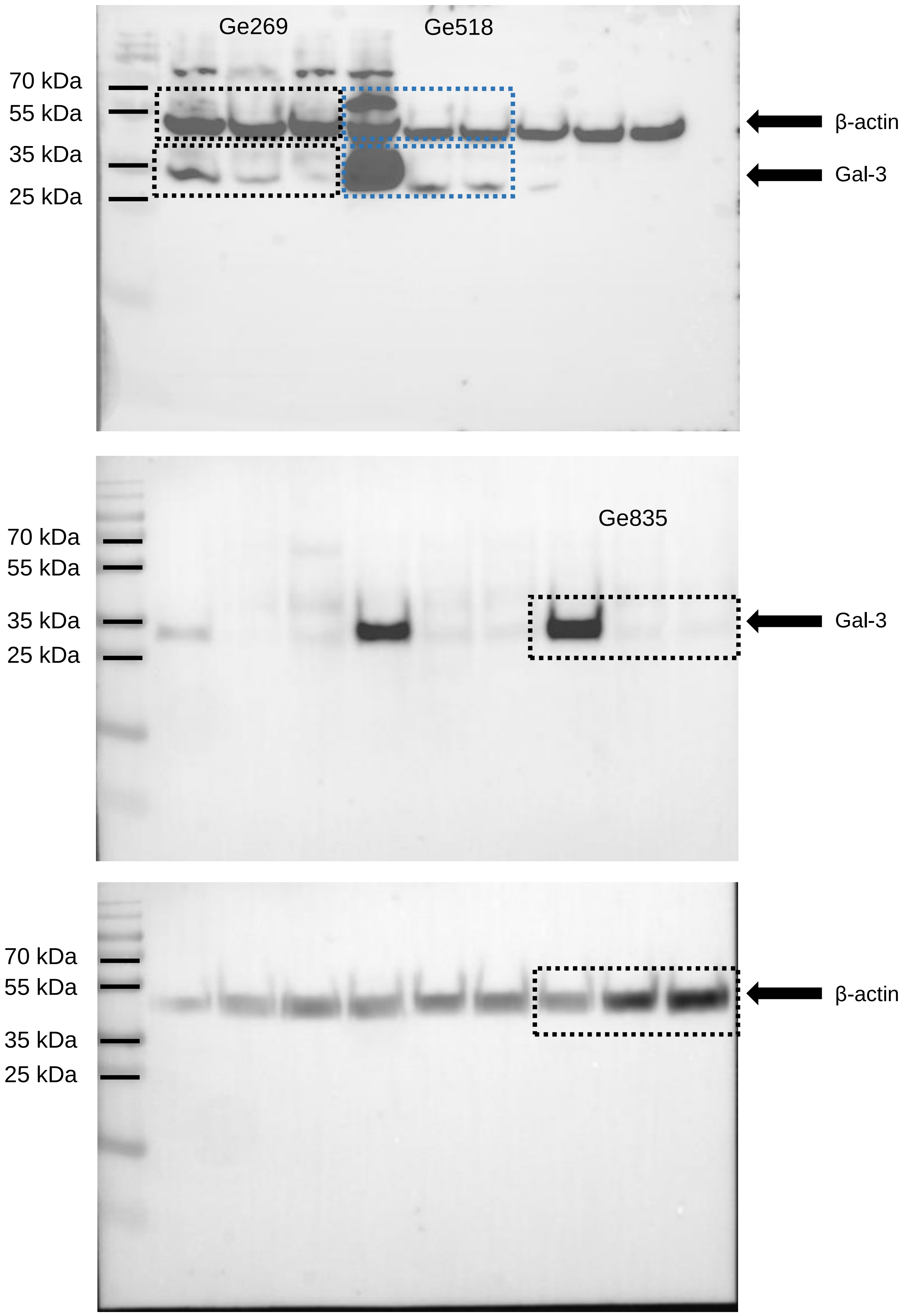

Supplementary Figure 10**.** The uncropped WB images showing the experiment displayed in the figures.

Figure 3D

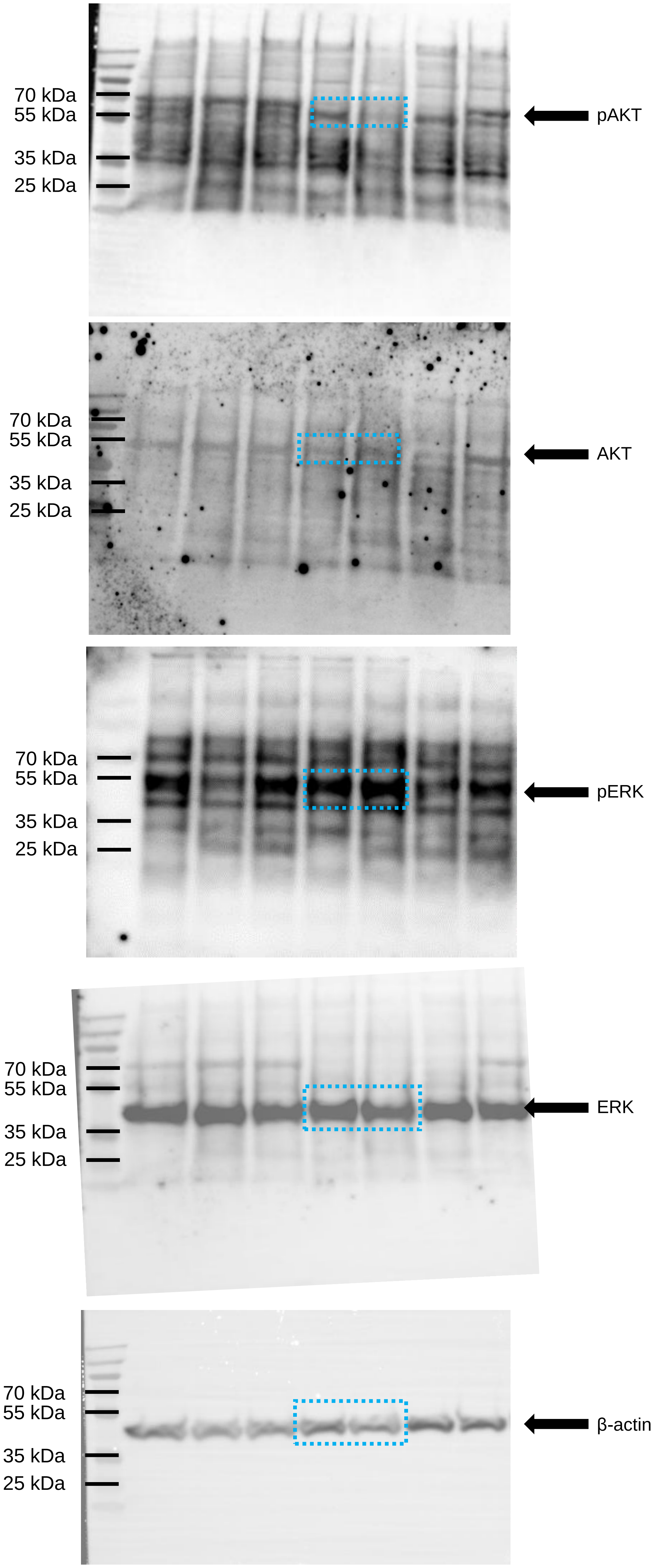

Supplementary Figure 10, *continued*

Figure 5C

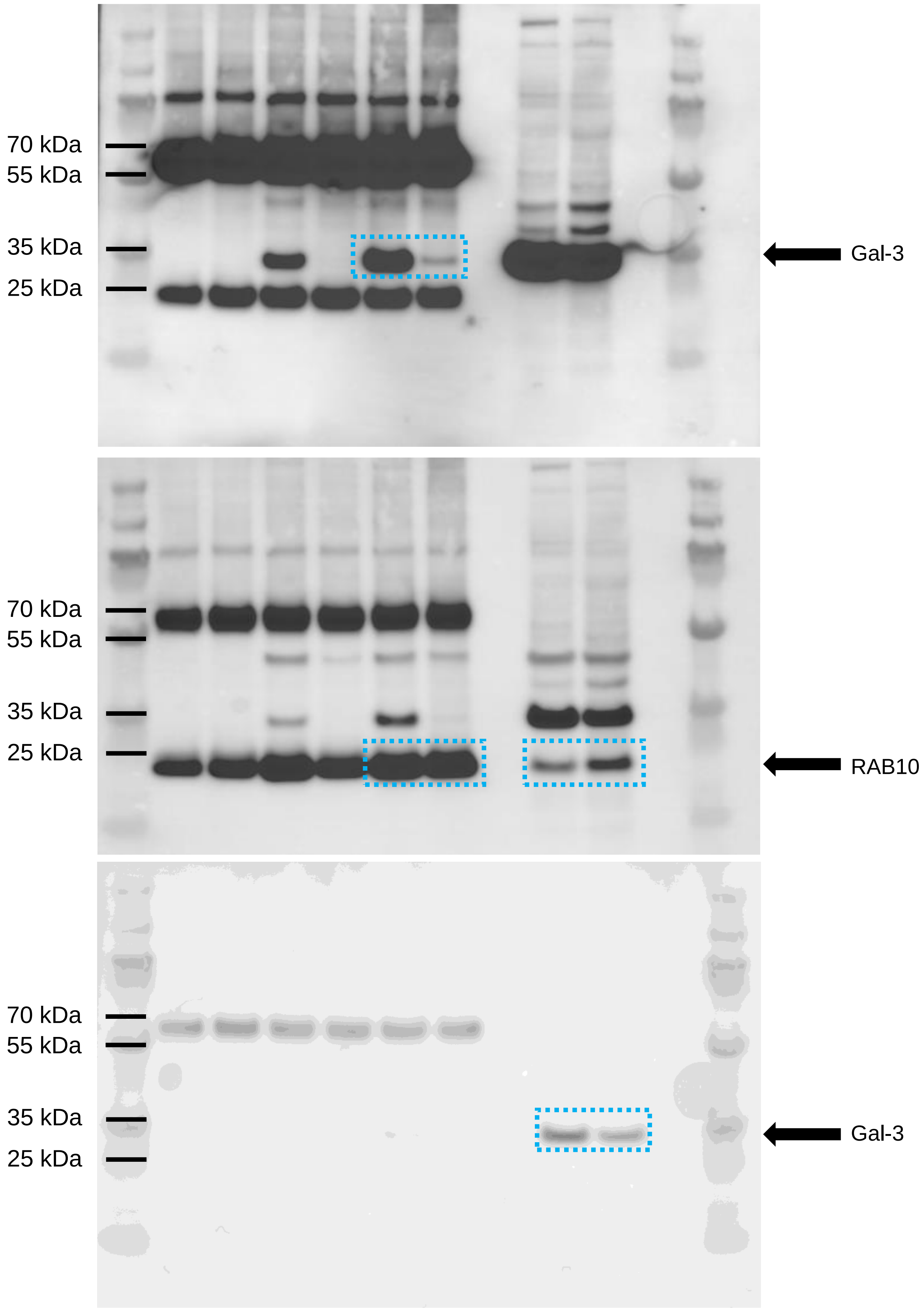

Supplementary Figure 10, *continued*

Figure 5D

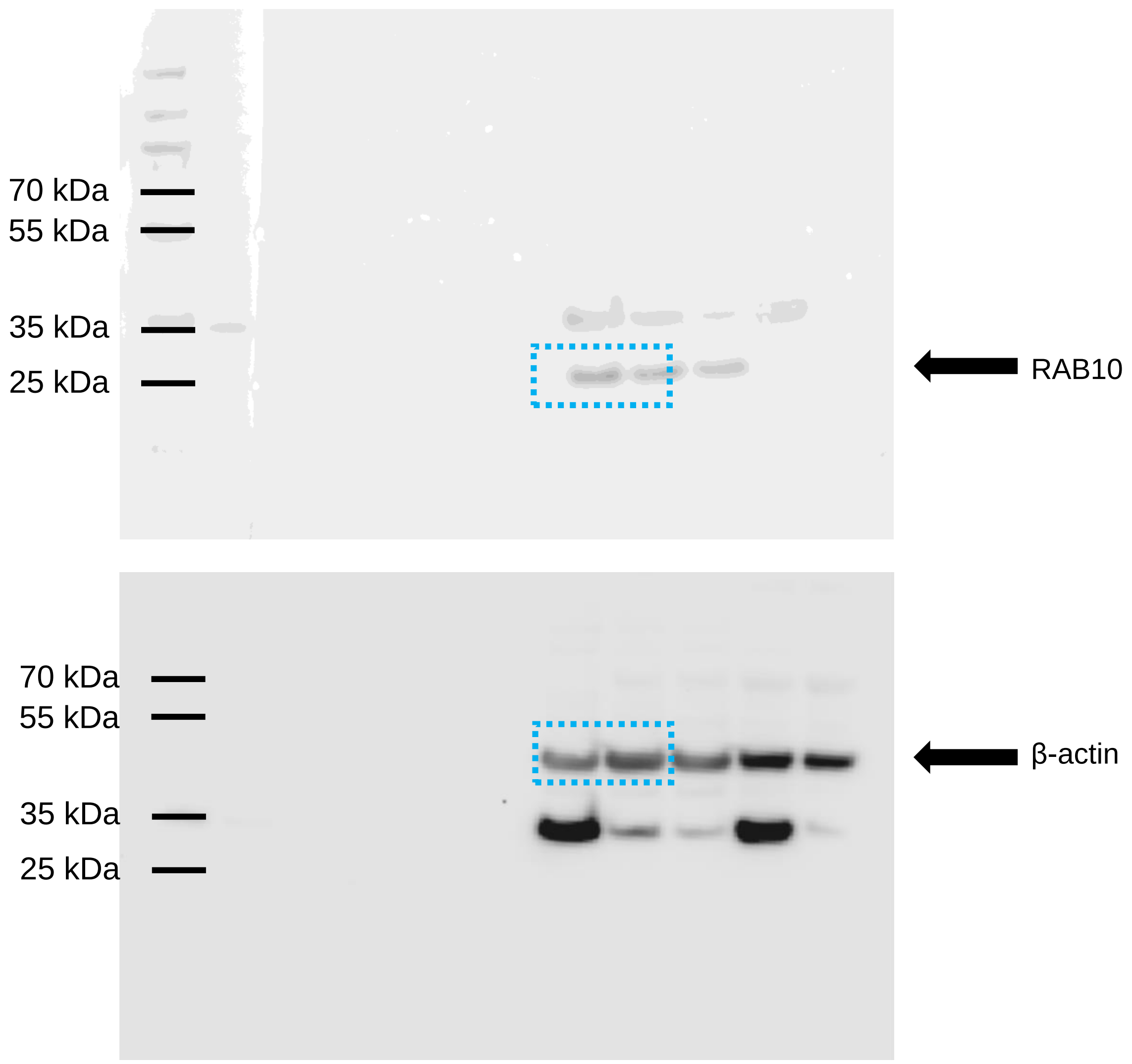

Supplementary Figure 10, *continued*

Figure 5F

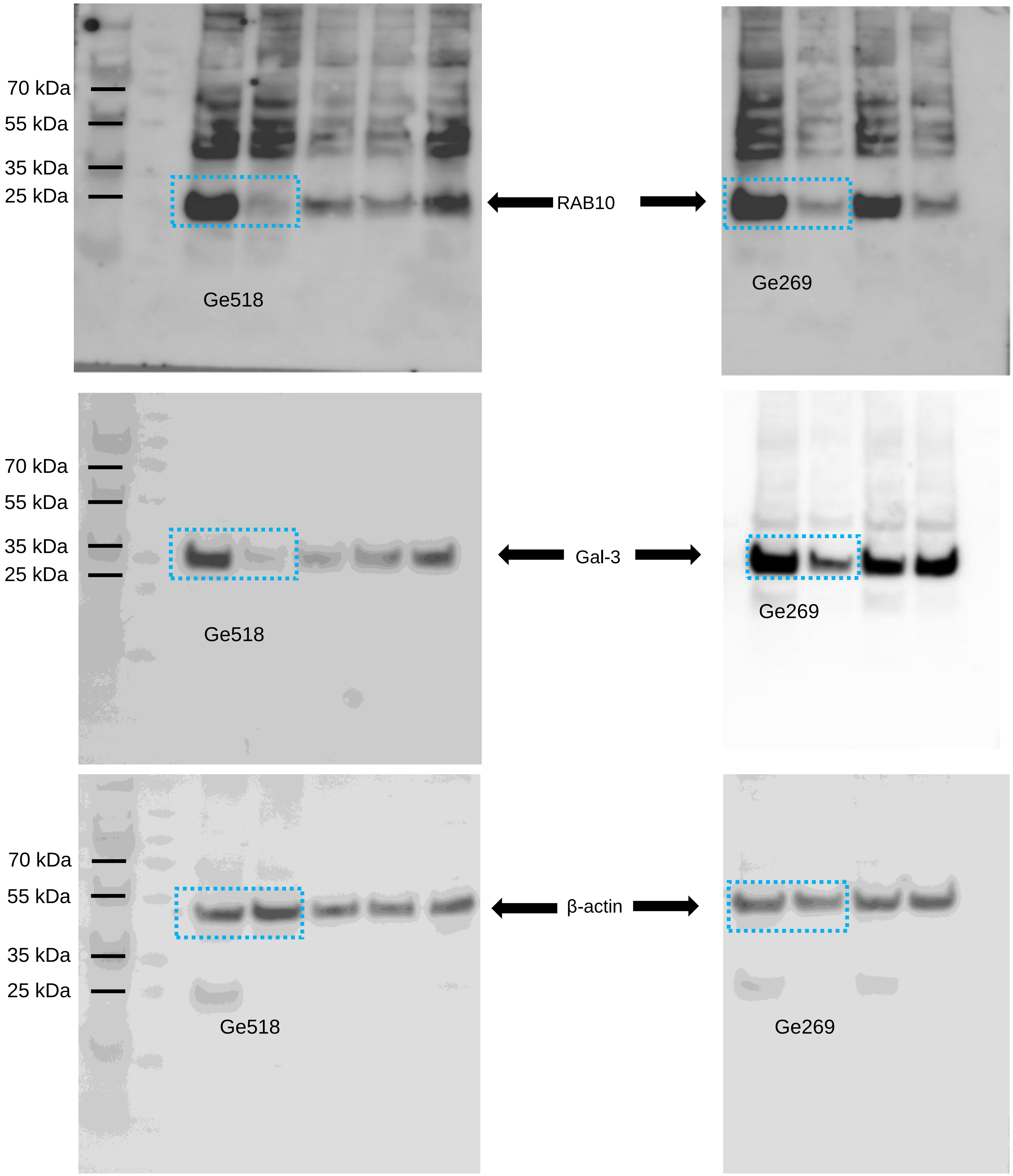

Supplementary Figure 10, *continued*

Suppl Figure 3A

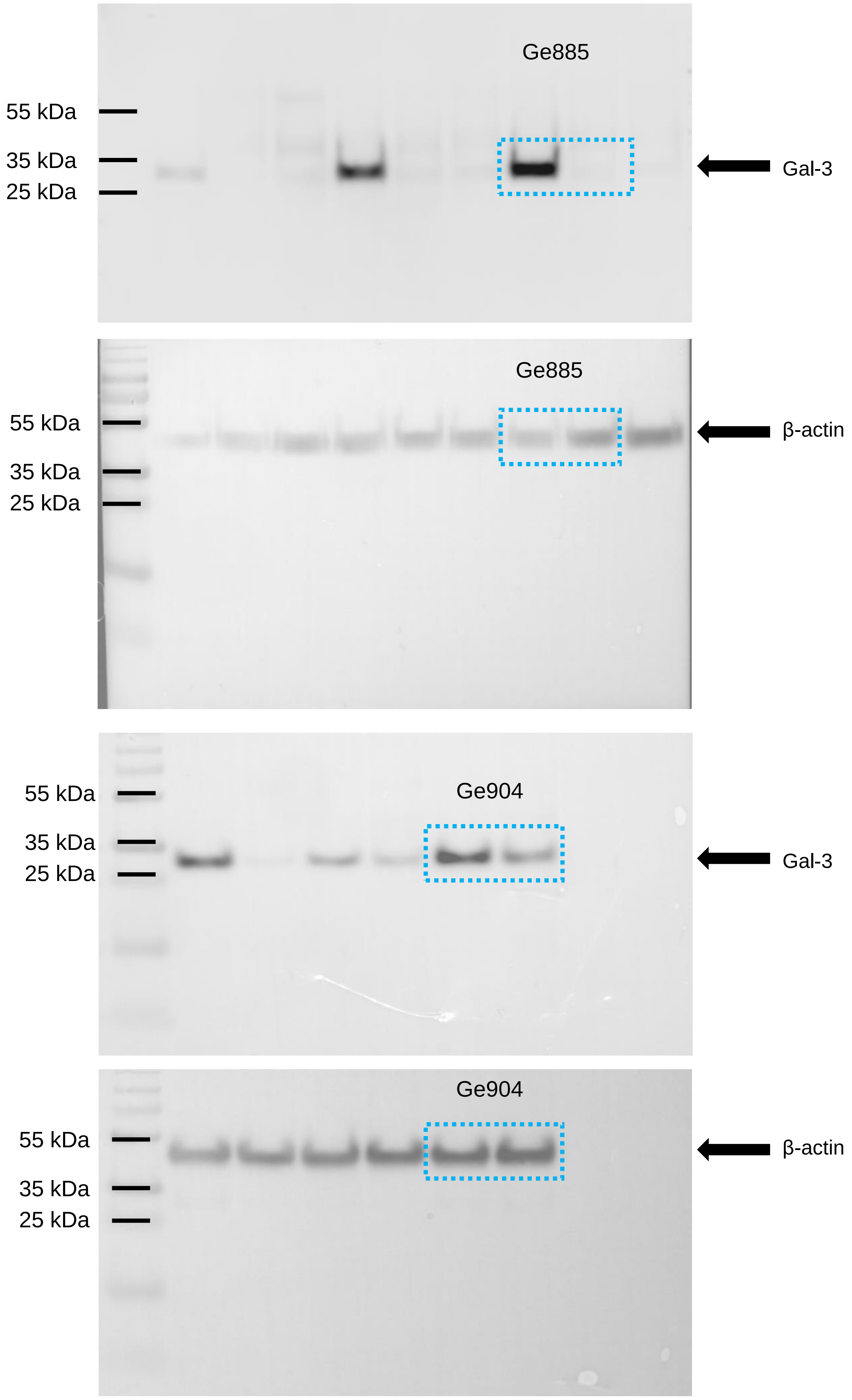

Supplementary Figure 10, *continued*

Suppl Figure 3E

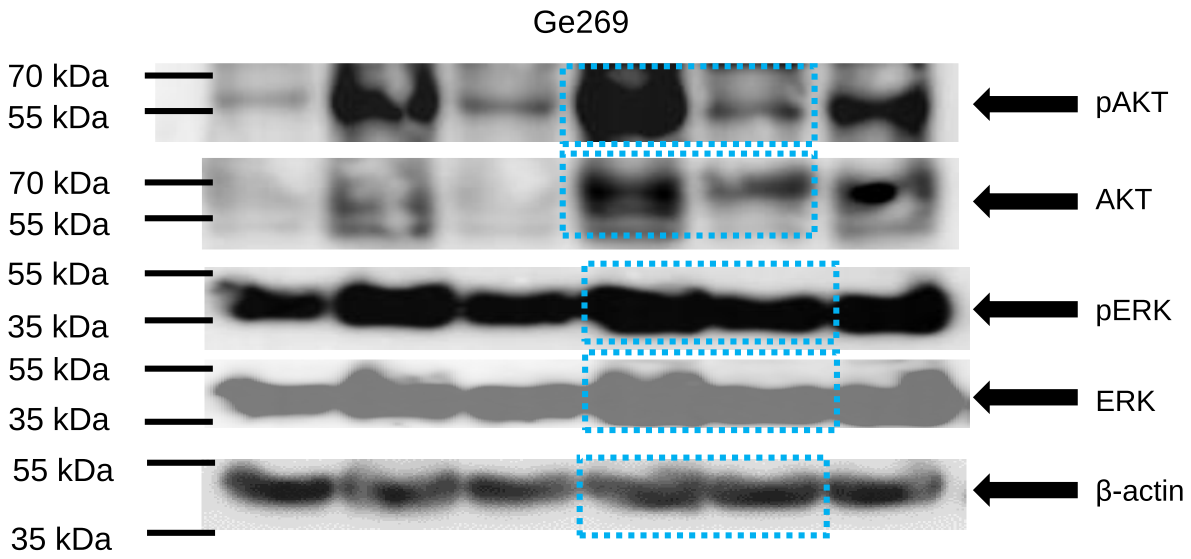

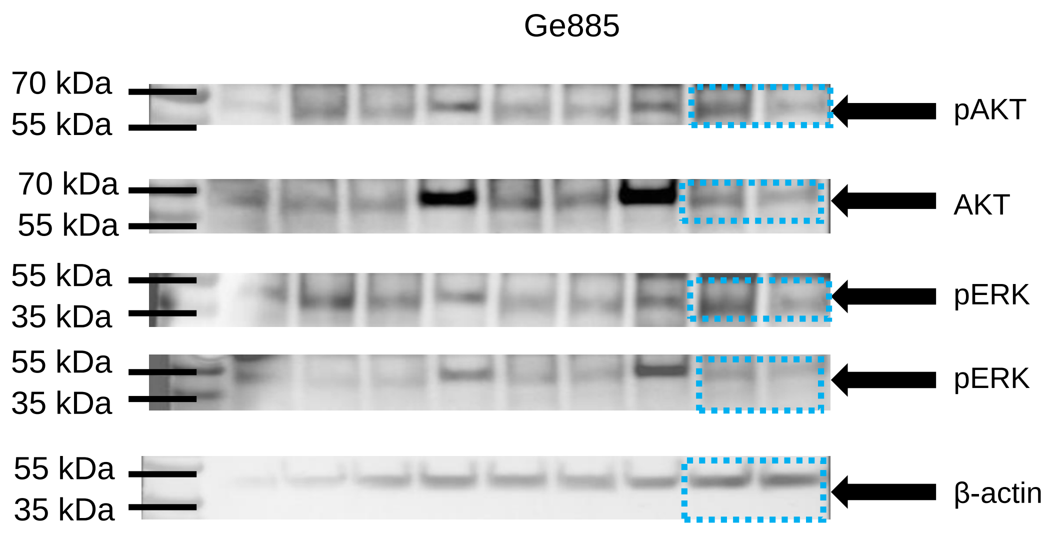

Supplementary Figure 10, *continued*

Suppl Figure 4C

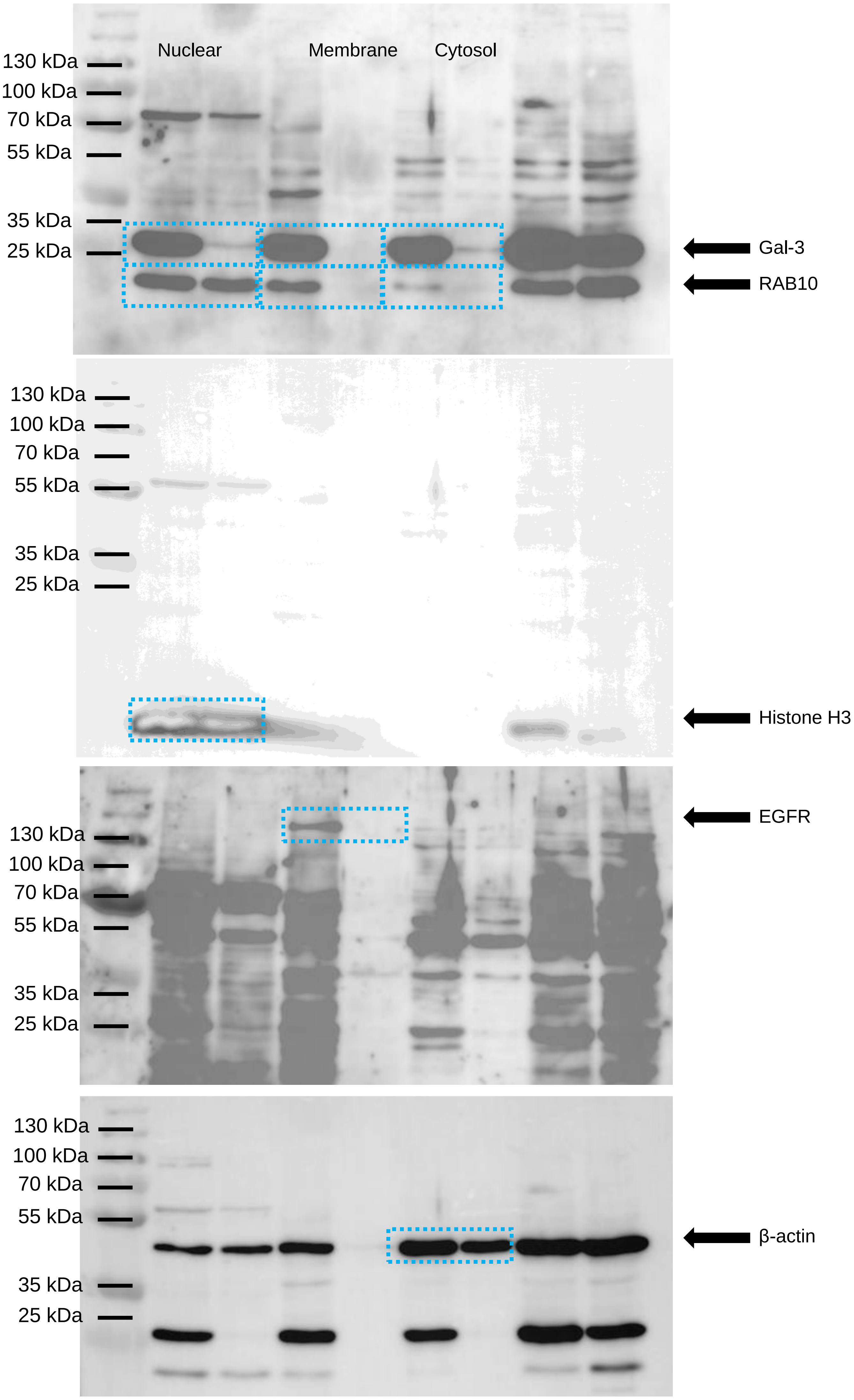

Supplementary Figure 10, *continued*

Suppl Figure 6B

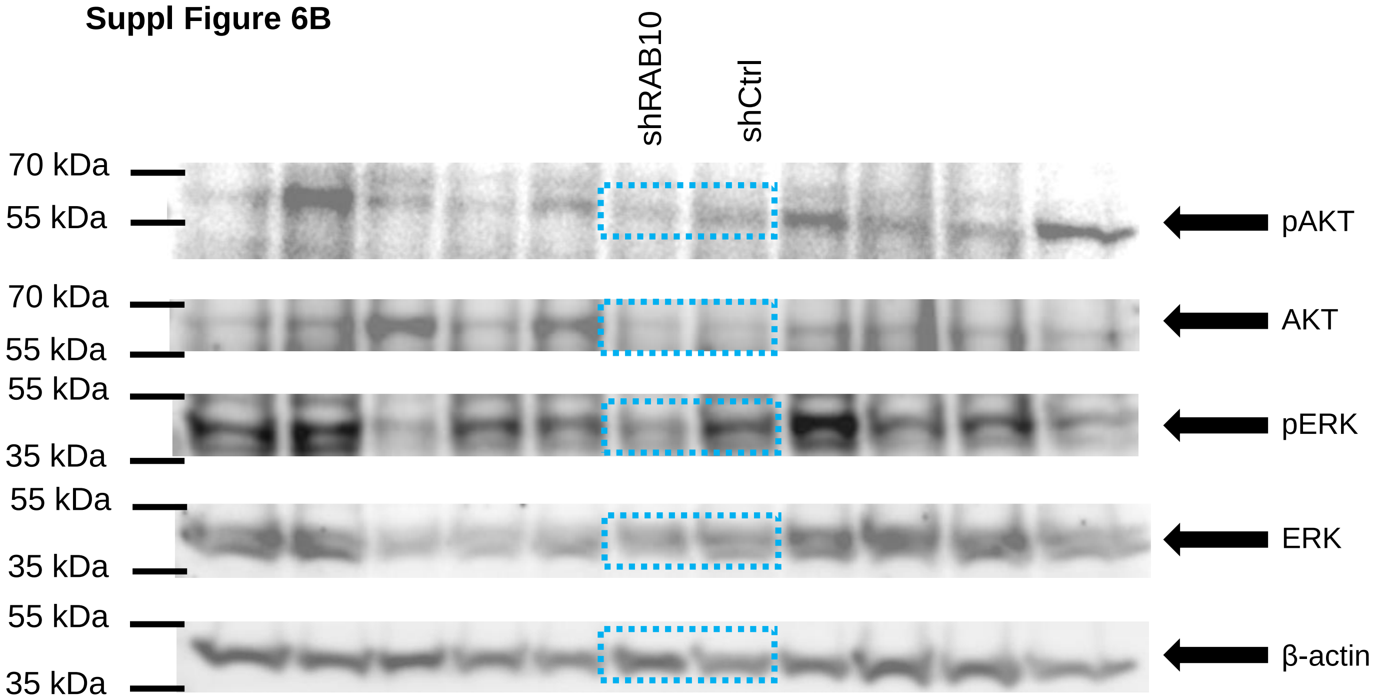

Supplementary Figure 10, *continued*

Suppl Figure 7A

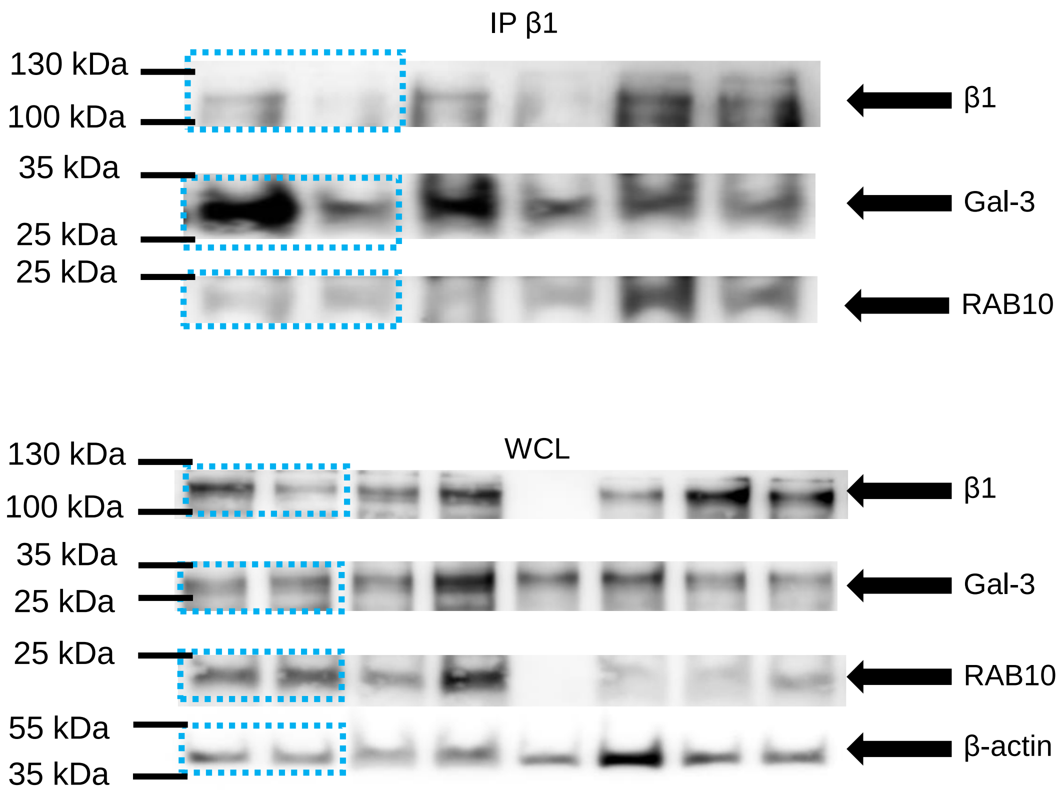

Supplementary Tables

	Log-rank pValue		
**Name**	**TCGA**	**Rembrandt**	**Gravendeel**
**LGALS1**	0.1357	0.5366	1.00E-04
**LGALS2**	0.8009	0.2986	0.8009
**LGALS3**	0.0134	0.0017	< 0.01
**LGALS4**	0.3993	0.0341	0.3685
**LGALS8**	0.0019	0.702	5.00E-04
**LGALS9**	0.0376	0.4427	0.0013
**LGALS12**	N/A	0.3662	0.0404
**LGALS13**	0.5443	0.0619	0.2935
**LGALS14**	0.0506	0.0815	0.4763

Supplementary Table 1. Gal-3 expression predicts poor survival among several GBM datasets (TCGA, Rembrandt, Gravendeel).

	TCGA							
	**Gal-3 addicted**	**Gal-3 non-addicted**		**Gal-3 addicted**	**Gal-3 non-addicted**		**Gal-3 addicted**	**Gal-3 non-addicted**
**1**	COL1A1	KLRC1	**31**	RBP1	FERMT1	**61**	CXCL8	TCEAL2
**2**	CHI3L1	TNR	**32**	TGFBI	GPR17	**62**	SLC2A10	APBA2
**3**	COL3A1	SCN3A	**33**	PDLIM4	DCX	**63**	COL5A1	CAMTA1
**4**	COL3A1	FUT9	**34**	CCL2	ALDOC	**64**	EMP1	TMEM100
**5**	COL1A2	OLIG2	**35**	LYZ	SCN3B	**65**	CYR61	CHD7
**6**	POSTN	TOX3	**36**	CYP1B1	KLRC3	**66**	COL1A1	FAM171A1
**7**	COL6A2	ETNPPL	**37**	CD163	ATP6V1G2	**67**	CD44	CLASP2
**8**	COL3A1	GRIA2	**38**	CA12	FXYD6	**68**	CD44	KCNQ2
**9**	COL1A2	SH3GL2	**39**	PDPN	RIMS3	**69**	COL4A1	SLC1A1
**10**	LGALS3	ASCL1	**40**	CA12	NMNAT2	**70**	S100A4	SATB1
**11**	COL6A3	DNM3	**41**	VEGFA	FGF13	**71**	GBP1	BAI3
**12**	TIMP1	GPR17	**42**	CD163	BASP1	**72**	COL5A2	EYA1
**13**	FABP5	SNAP91	**43**	CA12	STMN4	**73**	LAMB1	ELMO1
**14**	LTF	OPCML	**44**	ANXA2	ADAM22	**74**	MRC1	CUX2
**15**	LUM	COPG2IT1	**45**	COL5A1	WASF1	**75**	PDPN	PEG10
**16**	SERPINE1	TOX3	**46**	SERPING1	RTN1	**76**	CD44	KLRC2
**17**	GPNMB	DLL3	**47**	CYP1B1	PTCH1	**77**	WWTR1	MMP16
**18**	IGFBP2	PDGFRA	**48**	C1R	KCND2	**78**	THBS1	PAK3
**19**	PTX3	FERMT1	**49**	PLA2G5	SCG3	**79**	S100A11	CDH18
**20**	S100A8	TOX3	**50**	MGP	HMP19	**80**	CXCR4	FRY
**21**	NNMT	CDK5R1	**51**	LGALS1	ATP1A3	**81**	HSPA6	NET1
**22**	TAGLN	ELAVL4	**52**	NAMPT	ATP8A1	**82**	COL6A1	C1QL1
**23**	LOXL1	INA	**53**	DCN	BEX1	**83**	EFEMP2	GABBR2
**24**	EMP3	PLCB1	**54**	CYR61	DPYSL4	**84**	CA12	SEZ6L
**25**	DCN	OMG	**55**	VEGFA	ERC2	**85**	SDC2	MLLT11
**26**	ANXA1	LPPR1	**56**	COL4A2	SLITRK5	**86**	SLC16A3	CNTN1
**27**	LOX	SLIT1	**57**	TNFAIP6	ASCL1	**87**	IGFBP3	NRXN1
**28**	ADM	CLASP2	**58**	MYL9	RAB33A	**88**	CLIC1	STXBP1
**29**	SERPINE1	RUFY3	**59**	MMP9	MAPT	**89**	CD44	TMEM35
**30**	NAMPT	RTN1	**60**	LOX	BCAN	**90**	COL5A1	KCNN3

Supplementary Table 2. List of genes differentially expressed based on Gal-3^{high} *vs* Gal-3^{low} expression for the TCGA_GBM dataset. Only the top 120 genes are showed, ranked from 1 to 120. Only genes with adjusted p value <0.05 have been considered for analysis.

	HG-U133A		Gravendeel		Rembrant		Freije	
	pValue		pValue		pValue		pValue	
	Log-rank	Wilcoxon	Log-rank	Wilcoxon	Log-rank	Wilcoxon	Log-rank	Wilcoxon
**LOXL4**	0.129	0.0459	0.4702	0.8262	0.8657	0.2695	N/A	N/A
**CADM1**	<0.001	<0.001	0.1174	0.4763	0.3951	0.3955	0.1582	0.1683
**MGP**	0.129	0.0459	0.0015	0.0075	0.3685	0.4304	0.6314	0.7844
**NFATC2**	N/A	N/A	0.0491	0.0147	0.6638	0.6289	N/A	N/A
**S100A4**	0.0095	0.0291	<0.001	<0.001	0.6096	0.9047	0.4628	0.6777
**WNT5A**	0.0566	0.0552	<0.001	<0.001	<0.001	0.0012	0.2135	0.1373
**ITGB8**	0.003	0.0395	0.0015	<0.001	<0.001	<0.001	0.4459	0.5353
**COL8A1**	0.0161	0.0119	0.0291	<0.001	<0.001	<0.001	0.7429	0.8562
**PTPRZ1**	0.4833	0.4557	0.5186	0.9975	0.1143	0.0512	0.6928	0.9185
**PDGFA**	0.0067	0.0878	<0.001	<0.001	<0.001	<0.001	0.0069	0.0138
**ANXA2**	0.0967	0.0596	<0.001	<0.001	<0.001	<0.001	<0.001	<0.001
**ANXA1**	0.0032	0.0196	<0.001	<0.001	<0.001	<0.001	0.0277	0.043
**PTN**	0.547	0.8505	<0.001	<0.001	<0.001	<0.001	0.0054	0.0161
**CAV1**	0.2361	0.0502	<0.001	<0.001	<0.001	<0.001	0.3806	0.2759
**PTX3**	0.0184	0.1319	<0.001	<0.001	<0.001	<0.001	0.1391	0.1592
**CNTN1**	0.9733	0.4128	<0.001	<0.001	<0.001	<0.001	0.006	0.0295
**CAMKV**	0.4475	0.7367	0.0078	0.0125	<0.001	<0.001	0.5232	0.3134
**IGFBP5**	0.0031	0.0753	<0.001	<0.001	<0.001	<0.001	0.1135	0.1014
**CHI3L1**	0.0038	0.0194	<0.001	<0.001	<0.001	<0.001	0.0015	0.007

Supplementary Table 3. PDGFα, ANXA1, IGFBP5 and CHI3L1 expression predict poor survival among several GBM datasets (TCGA, Rembrandt, Gravendeel and Freije).

	Protein ID	# of physical associations		Protein ID	# of physical associations		Protein ID	# of physical associations		Protein ID	# of physical associations
**1**	ABCB1	2	**61**	FCF1	1	**121**	MYOC	1	**181**	SLC6A1	1
**2**	ABCC4	2	**62**	FCGR2A	1	**122**	NAP1L1	1	**182**	SLC7A2	2
**3**	ACP2	2	**63**	FLT4	2	**123**	NCSTN	1	**183**	SLC9A6	2
**4**	ACTA1	1	**64**	FN1	2	**124**	NID2	2	**184**	SLC12A2	3
**5**	ACTA2	1	**65**	FOXA1	1	**125**	NPTN	1	**185**	SLC12A4	2
**6**	ADCY3	2	**66**	GEMIN4	1	**126**	OPALIN	1	**186**	SLC12A6	2
**7**	ADCY6	2	**67**	GLB1	1	**127**	OSTM1	2	**187**	SLC12A7	2
**8**	ADCY9	2	**68**	GOLGA2	1	**128**	P2RY12	1	**188**	SLC14A1	1
**9**	AGPS	1	**69**	GPR35	2	**129**	PARP1	1	**189**	SLC15A4	2
**10**	AGR2	1	**70**	GPR52	2	**130**	PAXIP1	2	**190**	SLC17A5	1
**11**	AHCY	1	**71**	GPR55	2	**131**	PCBP2	1	**191**	SLC25A5	1
**12**	ALCAM	2	**72**	GPR84	1	**132**	PCCA	1	**192**	SLC26A2	2
**13**	APLNR	1	**73**	GRPR	1	**133**	PDCD1LG2	1	**193**	SLC30A1	2
**14**	APP	1	**74**	GTF2I	2	**134**	PDHX	1	**194**	SLC38A9	2
**15**	ATG9A	2	**75**	HEG1	2	**135**	PEBP1	1	**195**	SLC46A3	1
**16**	ATP1A1	1	**76**	HRNR	1	**136**	PLIN3	1	**196**	SOD1	1
**17**	ATP2B4	2	**77**	HSP90AB1	1	**137**	PODXL	2	**197**	SPR	1
**18**	ATP5C1	1	**78**	HTR2C	1	**138**	PPIG	1	**198**	SQSTM1	1
**19**	ATP13A3	2	**79**	ICOSLG	1	**139**	Ppp2r1a	1	**199**	SS18L1	1
**20**	BARD1	3	**80**	IGBP1	1	**140**	PRPF8	1	**200**	SYPL1	1
**21**	BRCA1	1	**81**	IL31RA	1	**141**	PRPF19	1	**201**	TACR1	1
**22**	BSG	1	**82**	IPO5	1	**142**	PRR13	1	**202**	TEX35	2
**23**	C1GALT1C1	1	**83**	IPPK	1	**143**	PTGFRN	2	**203**	TMEM9	1
**24**	C1orf85	1	**84**	ITGA1	2	**144**	PTPN11	2	**204**	TMEM63A	2
**25**	C11orf87	1	**85**	ITGA2	2	**145**	PTPRJ	2	**205**	TMEM63C	1
**26**	CACNG1	1	**86**	ITGB1	1	**146**	PTPRK	2	**206**	TMEM179B	1
**27**	CACNG5	1	**87**	KCTD12	1	**147**	PTPRO	1	**207**	TMEM182	1
**28**	CADM1	1	**88**	KIAA0319L	2	**148**	PTPRZ1	2	**208**	TMPO	1
**29**	CAPN1	2	**89**	KIAA1549	3	**149**	PYHIN1	2	**209**	TMSB10	1
**30**	CCT3	1	**90**	KPNB1	1	**150**	RAB7A	1	**210**	TPCN2	1
**31**	CD58	2	**91**	KRAS	1	**151**	RAB11B	1	**211**	TRIM5	2
**32**	CD63	2	**92**	LAMA1	2	**152**	RNF167	1	**212**	TRIM6	1
**33**	CD68	1	**93**	LAMA4	2	**153**	RPL7A	1	**213**	TRIM16	1
**34**	CD109	2	**94**	LAMB1	2	**154**	RPL12	1	**214**	TRIM17	1
**35**	CDC5L	1	**95**	LAMC1	1	**155**	RPL35	1	**215**	TRIM22	1
**36**	CDK15	1	**96**	LAMP1	1	**156**	RPS20	1	**216**	TRIM23	1
**37**	CFTR	1	**97**	LAMP2	1	**157**	RRAGB	2	**217**	TRIM49	2
**38**	CKAP4	1	**98**	LGALS1	1	**158**	RRAGC	2	**218**	TSPAN2	1
**39**	CLCN3	2	**99**	LGALS3BP	6	**159**	RTN4RL2	2	**219**	TSPAN31	1
**40**	CLCN5	1	**100**	LGALS3	1	**160**	SCARA3	2	**220**	TXN	1
**41**	CLNS1A	1	**101**	LGALS9C	2	**161**	SDK1	2	**221**	VIM	1
**42**	COASY	2	**102**	LGALS9	2	**162**	SDK2	2	**222**	VSTM1	1
**43**	COLEC12	2	**103**	LNPEP	2	**163**	SERINC1	1			
**44**	COX17	1	**104**	LPAR1	2	**164**	SERINC2	1			
**45**	CRIP1	1	**105**	LPAR2	1	**165**	SERPINH1	1			
**46**	CRYZ	1	**106**	LRP1	1	**166**	SGOL2	1			
**47**	CSPG4	2	**107**	MAP1LC3A	1	**167**	SLC1A3	1			
**48**	CTNNB1	1	**108**	MAPK3	1	**168**	SLC1A5	2			
**49**	CYLD	1	**109**	MCCC1	1	**169**	SLC2A14	1			
**50**	DBI	1	**110**	MCCC2	1	**170**	SLC3A2	1			
**51**	DDOST	1	**111**	MCPH1	1	**171**	SLC4A2	2			
**52**	ECE1	2	**112**	MEFV	1	**172**	SLC4A7	2			
**53**	EGFR	3	**113**	MFAP3	2	**173**	SLC5A5	1			
**54**	ELMOD1	1	**114**	MFSD4	1	**174**	SLC5A8	1			
**55**	ELN	2	**115**	MPZL1	2	**175**	SLC2A14	1			
**56**	EMB	2	**116**	MRC2	2	**176**	SLC3A2	1			
**57**	EMP3	1	**117**	MYH10	1	**177**	SLC4A2	2			
**58**	ENPP4	2	**118**	MYL6	1	**178**	SLC4A7	2			
**59**	ESR2	1	**119**	MYL12B	1	**179**	SLC5A5	1			
**60**	FBXL4	1	**120**	MYO1E	1	**180**	SLC5A8	1			

Supplementary Table 4. List of proteins that physically interact with Gal-3 (from Biogrid https://thebiogrid.org/).

RPPA TCGA_GBM (Agilent 4502A)					
**nb**	**Protein name**	**nb**	**Protein name**	**nb**	**Protein name**
**1**	PAI.1	**31**	FoxM1	**61**	NF2
**2**	IGFBP2	**32**	c.Jun_pS73	**62**	Paxillin
**3**	Caveolin.1	**33**	p62.LCK.ligand	**63**	PR
**4**	Collagen_VI	**34**	Bad_pS112	**64**	LKB1
**5**	CDK1_pY15	**35**	VEGFR2	**65**	p90RSK
**6**	MSH2	**36**	ACC_pS79	**66**	mTOR_pS2448
**7**	Myosin.IIa_pS1943	**37**	Dvl3	**67**	PDK1_pS241
**8**	TFRC	**38**	p70S6K_pT389	**68**	FOXO3a
**9**	Bap1.c.4	**39**	HER2_pY1248	**69**	Annexin_VII
**10**	Fibronectin	**40**	14.3.3_beta	**70**	Cyclin_B1
**11**	ER.alpha_pS118	**41**	Stathmin	**71**	p38_pT180_Y182
**12**	Annexin.1	**42**	14.3.3_epsilon	**72**	EGFR_pY1173
**13**	c.Kit	**43**	Chk1_pS345	**73**	Mre11
**14**	TAZ	**44**	Bim	**74**	Bcl.2
**15**	p21	**45**	MIG.6	**75**	PEA15
**16**	Rictor	**46**	P.Cadherin	**76**	BRCA2
**17**	ACC1	**47**	JNK2	**77**	Rab25
**18**	Bax	**48**	SCD	**78**	Rb
**19**	Chk2	**49**	4E.BP1_pS65	**79**	Chk1
**20**	YB.1	**50**	DJ.1	**80**	MAPK_pT202_Y204
**21**	ARID1A	**51**	PARP_cleaved	**81**	RBM15
**22**	GSK3.alpha.beta	**52**	4E.BP1	**82**	EGFR_pY1068
**23**	p27	**53**	p70S6K		
**24**	HER2	**54**	HSP70		
**25**	Src_pY527	**55**	Tuberin_pT1462		
**26**	CD49b	**56**	HER3_pY1289		
**27**	ARHI	**57**	GSK3_pS9		
**28**	ACVRL1	**58**	SF2		
**29**	PDK1	**59**	Caspase.8		
**30**	E.Cadherin	**60**	B.Raf		

Supplementary Table 5. The reverse phase protein array for Gal-3 from TCGA dataset.

GBM	TCGA SUBTYPE	EGFR AMP.	EGFR MUT.	MGMT METHYLATION	TERT	IDH1	IDH2	EGRF	TP53	BRAF	HRAS	KIT	MET	HRAS2	NRAS	PDGFRA	PIK3CA	PTEN	PTPN11
6	C	Y	VIII	U	C228T	wt	wt		mt										
10	M	N		U	C228T	wt	wt												
12	N	Y		M	C250T	wt	wt	mt											
39	M	Y	VIII	M	C250T	wt	wt											mt	
59	M	Y	VIII	M	C228T	wt	wt												
64	P	Y		U	C228T	wt	wt									mt			mt
116	M			M	C228T	wt	wt												
150	M			U	C228T	fail	wt												
Ge518	M		wt			wt	wt						mt						
Ge269	M					wt	wt												
Ge835	M		wt	M		wt	wt												
Ge885	N																		
Ge898	P					wt	wt												
Ge904	C					wt	wt												
Ge970.2	C																		

Supplementary Table 6. Mutations found in GSCs.

#	Name	Sequence	#	Name	Sequence
1	**Cav1 FWD**	ACCGCGACCCTAAACACCTC	51	**SH3GL2 FWD**	ACTGTCGGAGGTCAAAGACTC
2	**Cav1 REV**	CCTTCCAAATGCCGTCAAAA	52	**SH3GL2 REV**	CCGGAATCTTGCCTTGTCGTT
3	**PCNA FWD**	GGCTCTAGCCTGACAAATGC	53	**DCX FWD**	GACAGCCCACTCTTTTGAGC
4	**PCNA REV**	GCCTCCAACACCTTCTTGAG	54	**DCX REV**	TGGGTTTCCCTTCATGACTC
5	**ITGB8 FWD**	ACCAGGAGAAGTGTCTATCCAG	55	**LOX FWD**	CCTACTACATCCAGGCGTCCA
6	**ITGB8 REV**	CCAAGACGAAAGTCACGGGA	56	**LOX REV**	CATAATCTCTGACATCTGCCCCTGT
7	**LOXL4 FWD**	CTGGGCACCACTAAGCTCC	57	**CD44 FWD**	AAGGTGGAGCAAACACAACC
8	**LOXL4 REV**	CTCCTGGATAGCAAAGTTGTCAT	58	**CD44 REV**	AGCTTTTTCTTCTGCCCACA
9	**PTPRZ1 FWD**	GCCTGGATTGGGCTAATGGAT	59	**ETNPPL FWD**	AAGTGGGCTTTGGCAGAGTT
10	**PTPRZ1 REV**	CAGTGCTCCTGTATAGGACCA	60	**ETNPPL REV**	TGCCCATCGGTTTTCCCATT
11	**PTN FWD**	GGAGCTGAGTGCAAGCAAAC	61	**PLA2G5 FWD**	CTGGCTCCAGTTGGAACACT
12	**PTN REV**	CTCGCTTCAGACTTCCAGTTC	62	**PLA2G5 REV**	GAGAAAGAGGCACCAGCGAT
13	**COL8A1 FWD**	GGGAGTGCTGCTTACCATTTC	63	**ITGB5 FWD**	TCTCGGTGTGATCTGAGGG
14	**COL8A1 REV**	AGCGGCTTGATCCCATAGTAG	64	**ITGB5 REV**	TGGCGAACCTGTAGCTGGA
15	**CADM1 FWD**	ATGGCGAGTGTAGTGCTGC	65	**ITGB1 FWD**	TGTAACCAACCGTAGCAAAGGA
16	**CADM1 REV**	GATCACTGTCACGTCTTTCGT	66	**ITGB1 REV**	CCCTGATCTTAATCGCAAAACC
17	**MGP FWD**	TCCGAGAACGCTCTAAGCCT	67	**ITGB3 FWD**	GTGACCTGAAGGAGAATCTGC
18	**MGP REV**	GCAAAGTCTGTAGTCATCACAGG	68	**ITGB3 REV**	TCACTCACTGGGAACTCGATG
19	**NFATC2 FWD**	GAGCCGAATGCACATAAGGTC	69	**ITGAV FWD**	AAGCTGAGCTCATCGTTTCC
20	**NFATC2 REV**	CCAGAGAGACTAGCAAGGGG	70	**ITGAV REV**	GCACAGGAAAGTCTTGCTAAGG
21	**PDGFA FWD**	GACACGGATGTGAGGTGAGG	71	**ITGA5 FWD**	GGGCAGCAGGACAGGGTTAC
22	**PDGFA REV**	GGAGGAGAACAAAGACCGCA	72	**ITGA5 REV**	GCCTTGCCAGAAATAGCTTCC
23	**WNT5A FWD**	GCCAGTATCAATTCCGACATCG	73	**ITGA4 FWD**	CACAACACGCTGTTCGGCTA
24	**WNT5A REV**	TCACCGCGTATGTGAAGGC	74	**ITGA4 REV**	CGATCCTGCATCTGTAAATCGC
25	**IGFBP5 FWD**	ACCTGAGATGAGACAGGAGTC	75	**TOX3 FWD**	ATTCCACCAATCACGCCTCC
26	**IGFBP5 REV**	GTAGAATCCTTTGCGGTCACAA	76	**TOX3 REV**	GGATCGCTGAGGGCTTGAAA
27	**CNTN1 FWD**	CAGCCCTTTCCCGGTTTACAA	77	**EEF1A1 FWD**	AGCAAAAATGACCCACCAATG
28	**CNTN1 REV**	TGCTTCTGACCATCCCGTAGT	78	**EEF1A1 REV**	GGCCTGGATGGTTCAGGATA
29	**CHI3L1 FWD**	CAGAGTCGCCGGACTTTCAT	79	**ALAS1 FWD**	CTCACCACACACCCCAGATG
30	**CHI3L1 REV**	TGTCTCTCCGTCCAGGGTAG	80	**ALAS1 REV**	AGTTCCAGCCCCACTTGCT
31	**S100A4 FWD**	GATGAGCAACTTGGACAGCAA	81	**TBP FWD**	GCCCGAAACGCCGAATATA
32	**S100A4 REV**	CTGGGCTGCTTATCTGGGAAG	82	**TBP REV**	CGTGGCTCTCTTATCCTCATGA
33	**CAMKV FWD**	GATTTGGGACAGGTCATCAAGAC	83	**TUBA2 FWD**	AGGAGCTGGCAAGCATGTG
34	**CAMKV REV**	AGCTGTAGGATGTTGGGATGC	84	**TUBA2 REV**	CGGTGCGAACTTCATCGAT
35	**ALDH1A3 FWD**	ACCTCTCACCGCCCTTTATCT			
36	**ALDH1A3 REV**	GTGAAGGCGATCTTGTTGATCT			
37	**LGALS3 FWD**	GTGAAGCCCAATGCAAACAGA			
38	**LGALS3 REV**	AGCGTGGGTTAAAGTGGAAGG			
39	**ANXA1 FWD**	GCGGTGAGCCCCTATCCTA			
40	**ANXA1 REV**	TGATGGTTGCTTCATCCACAC			
41	**ANXA2 FWD**	GAGCGGGATGCTTTGAACATT			
42	**ANXA2 REV**	TAGGCGAAGGCAATATCCTGT			
43	**PTX3 FWD**	CATCTCCTTGCGATTCTGTTTTG			
44	**PTX3 REV**	CCATTCCGAGTGCTCCTGA			
45	**ELAVL4 FWD**	AACCTCTATGTTAGCGGCCTT			
46	**ELAVL4 REV**	TGGACACTCCTGTGACTTGAT			
47	**PLA2G5 FWD**	AAGGAGGCTTGCTGGACCTAA			
48	**PLA2G5 REV**	GCGAATGTTGCAGCCCTTC			
49	**SCN3A FWD**	CTCAGAAACCCATACCTCGCC			
50	**SCN3A REV**	CGGGACAAAACTAGGGTCATGTA			

Supplementary Table 7. List of primers used for qRT-PCR.
